# Unraveling the Effects of Freezing and Frozen Storage Temperatures on Hop Secondary Metabolites and Antioxidants

**DOI:** 10.3390/antiox15030310

**Published:** 2026-02-28

**Authors:** Bilge Ece Özel, Simona Tatasciore, Veronica Santarelli, Luca Valbonetti, Paola Pittia, Lilia Neri

**Affiliations:** Department of Bioscience and Technologies for Food Agriculture and Environment, University of Teramo, Via Renato Balzarini 1, 64100 Teramo, Italy; beozel@unite.it (B.E.Ö.); statasciore@unite.it (S.T.); vsantarelli@unite.it (V.S.); lvalbonetti@unite.it (L.V.); ppittia@unite.it (P.P.)

**Keywords:** hops, freezing and frozen storage, bitter acids, polyphenols, antioxidant properties

## Abstract

This study evaluated the effect of freezing and frozen storage at three temperatures (−20, −30, −40 °C) on hop (*Humulus lupulus* L.) secondary metabolites and antioxidant capacity. These temperatures were selected based on the glass transition temperature (T_g_’) of the maximally freeze-concentrated matrix. Cones were analyzed after freezing (t_0_) and up to 360 days (t_360_) by high-performance liquid chromatography with ultraviolet diode-array detection (HPLC-UV/DAD) for bitter acids, prenylflavonoids and phenolic acids, and by the Folin–Ciocalteu, ABTS the radical cation scavenging assay (ABTS) and the ferric-reducing antioxidant power assay (FRAP) assays for total phenolic content and antioxidant activity. Confocal laser scanning microscopy (CLSM) at t_360_ was used to relate microstructural damage to metabolite retention. Freezing at −40 °C ensured the highest retention of bitter acids, phenolic acids (gallic, syringic, vanillic, caffeic, chlorogenic), and antioxidant capacity, whereas xanthohumol and 8-prenylnaringenin reached their maximum levels at −30 and −20 °C, respectively. During frozen storage, changes in metabolite profiles were mainly driven by storage time rather than temperature; over 360 days, α-acids, colupulone, xanthohumol and selected phenolic acids increased, while most other compounds declined. Multivariate analysis and CLSM elucidated the relationships between process conditions, tissue structure and metabolite profiles, enabling the selection of freezing and storage temperatures to optimally preserve different targets of hop bioactives and overall indicating −40 °C as the most effective.

## 1. Introduction

In recent years, the food and nutraceutical industries have increasingly focused on identifying plant-based raw materials such as herbs, spices, and other officinal plants characterized by their distinctive functional and techno-functional properties. Among these, the female inflorescences of *Humulus lupulus* L., commonly known as hop cones, are particularly remarkable for their abundance of secondary metabolites with these characteristics [1]. Specifically, hop cones are morphologically composed of a central strig surrounded by overlapping bracts and bracteoles (leaf-like structures), which enclose numerous lupulin glands. These glands are biconical glandular trichomes, enveloped by the cuticle and subcuticular wall, that serve as the primary biosynthetic and storage sites for key hop secondary metabolites [2]. At maturity [3], these structures are filled with secreted compounds, primarily resins (bitter acids), prenylflavonoids, and essential oils [4].

Bitter acids are prenylated derivatives of phloroglucinol and are classified into α- and β-acids. The α-acids comprise a mixture of six humulone analogs, with n-humulone, cohumulone, and adhumulone being the most abundant, whereas the β-acids primarily include n-lupulone, colupulone, and adlupulone [5]. Prenylflavonoids, which accumulate mainly on the outer surface of the lupulin glands [6], constitute another key class of hop secondary metabolites. The major representatives include xanthohumol, isoxanthohumol, desmethylxanthohumol, and 6- and 8-prenylnaringenin. Other classes of phenolic compounds, including flavonols, flavan-3-ols, benzoic and cinnamic acids and their derivatives, are mainly present in the bracts and bracteoles [7].

Such molecules display a broad range of techno-functional activities, including sensory, antioxidant, and antimicrobial effects. Moreover, from a nutraceutical perspective, hop bioactives have been associated with potential benefits related to oxidative stress modulation, anti-inflammatory, antimutagenic, cardio and neuroprotective effects, making this plant an attractive source for the nutraceutical, pharmaceutical and food sectors beyond the brewing sector [8,9].

Despite their rich phytochemical profile, however, fresh hop cones are highly perishable due to their high moisture content and the rapid onset of enzymatic and chemical oxidation reactions. Consequently, post-harvest stabilization processes must be promptly adopted to preserve both the quality and techno-functional properties of hop cones. Industrially, immediately after harvest, the cones are typically subjected to hot air drying and eventually pelletized to facilitate handling and storage. These treatments effectively enhance product stability by lowering moisture content and water activity; however, they can also alter the native distribution of lupulin glands and compromise heat-sensitive metabolites, potentially reducing their concentration and properties [10]. In this framework, freezing represents a promising alternative stabilization strategy, potentially limiting oxidative and enzymatic degradation while maintaining a metabolite profile closer to that of fresh cones.

Freezing represents an effective preservation strategy, which combines the inhibitory action of low temperatures with the immobilization of water through ice crystallization, thereby limiting its availability as both solvent and reactive component. Lowering the temperature below the product’s freezing point inhibits post-harvest metabolic activity and slows microbial growth, as well as diffusion-controlled deterioration reactions. These effects primarily arise from the significant reduction in molecular mobility, which results from the decreased kinetic energy of reactants, water immobilization through crystallization, and increased viscosity of the unfrozen phase. However, freezing can exert controversial effects on oxidation and enzymatic pathways. The freeze-concentration of the unfrozen matrix, in fact, increases local reactant concentrations and alters key chemical properties (e.g., pH, ionic strength and redox environment), while low temperatures increase the oxygen solubility in the system; thus, the balance between these factors and the reduced molecular diffusivity ultimately governs the net reaction rate. In addition, the morphology and distribution of ice crystals can compromise cellular integrity by inducing membrane rupture and structural disruption phenomena that may adversely affect product quality [11]. Therefore, selection of both freezing and frozen storage temperature plays a central role in determining the physical stability, chemical integrity and long-term quality of frozen plant matrices. Freezing conditions regulate supercooling, ice nucleation and crystal growth, thereby affecting crystal size and mechanical damage. Specifically, lower temperatures/higher freezing rates typically promote smaller, more uniformly distributed crystals, reducing membrane rupture and the release and oxidation of sensitive bioactive compounds [12]. Moreover, freezing temperature influences the amount of freezable water and the physical state and chemical stability of the unfrozen phase. The selection of the frozen storage temperature is equally critical and should be defined based on the thermal properties of the product. When storage is conducted below the glass transition temperature of the maximally freeze-concentrated matrix (T_g_’), the unfrozen phase is characterized by extremely low molecular mobility. Under these conditions, diffusion-limited processes, including oxidation, enzyme-mediated degradation, phase separation and ice recrystallisation, are noticeably suppressed, thereby enhancing long-term stability [13,14]. Therefore, freezing and frozen storage temperatures cannot be selected empirically but must be rationally designed based on the specific T_g_’ of the food matrix, which in turn depends on the molecular weight distribution of its components, water-binding ability, solute interactions, and the structural organization of the tissue.

Despite the increasing industrial interest in frozen hop products, scientific studies regarding the impact of freezing conditions and frozen storage on the chemical stability of hop bioactives remain scarce. Existing studies, in fact, have mainly explored freezing as a pre-treatment in hop processing [15] or have considered variations in essential oil composition when cones are frozen before/after drying [16], rather than systematically addressing the effects on the structural damage, the antioxidant properties and the retention of secondary metabolites under extended frozen storage.

To address this knowledge gap, the present study the effect of freezing and frozen storage at three temperatures (−20, −30, −40 °C), selected based on hops’ thermal properties (T_g_’), on the secondary-metabolite profile of hop cones, focusing on their stability in relation to the physical state of the freeze-concentrated matrix. Microstructure analysis by CLSM (lupulin glands, bracts and bracteoles) was also performed to highlight structural damage induced by frozen storage at different temperatures.

## 2. Materials and Methods

### 2.1. Materials

Hop cones (*Humulus lupulus* cv. Cascade) were provided by a local producer in the Abruzzo region (Italy) and used for all the experiments. All used reagents were purchased from Sigma-Aldrich (Steinheim, Germany).

### 2.2. Thermal Properties and Freezable Water Determination

Thermal analysis of the hop cones was performed using a differential scanning calorimeter (DSC 8500, PerkinElmer, Waltham, MA, USA). For the analysis, ground bracts and bracteoles of the hop cones were used.

Instrument calibration was performed using indium. Hop cone samples, which were accurately weighed (ca. 7–10 mg), were placed into DSC aluminum pans (50 μL, PerkinElmer) and hermetically sealed in aluminum lids. An empty aluminum pan was used as a reference for baseline correction. Samples were cooled down from 20 °C to −80 °C at 5 °C/min, held isothermally for 5 min, and then heated back to the initial temperature at the same rate. The T_g_’ was identified as the onset point of the step change in heat flow during the second heating cycle, calculated automatically using Pyris™ software (version 11, PerkinElmer).

The amount of freezable water (g·g_fw_^−1^) was determined using

(1) $$X_{W}^{F}=\frac{\Delta H}{{\Delta H}_{ice}}$$

where ΔH (J/g) is the measured latent heat of melting of water per gram of sample, obtained by the integration of the melting endothermic peak; ΔH_ice_ (334 J/g) is the latent heat of melting of pure water at 0 °C [17].

### 2.3. Experimental Plan

Fresh hop cones are highly perishable and may undergo rapid post-harvest changes due to their high moisture content and ongoing metabolic activity. To minimize uncontrolled variability during handling, cones were transported to the laboratory and processed within approximately 2 h from harvest. Immediately upon arrival, samples were randomly portioned (≈50 g) and packed in polypropylene freezer bags, arranged as a single layer to promote uniform heat transfer. Thus, packages were distributed in three laboratory freezers set at −20 (static freezer; Whirlpool, Pero, Italy), −30 (no frost freezer; Miele, Gütersloh, Germany) and −40 °C (direct-freeze, deep freezer; NUVE, Ankara, Turkey), in which samples were both frozen and subsequently stored. For each temperature and storage time, four independent bags (n = 4) were prepared. Frozen storage was carried out for up to 360 days, and sampling was performed at predefined times, i.e., immediately after freezing (t_0_, experimental baseline for all subsequent comparisons) and after 7, 14, 30 and 360 days of storage (t_7_, t_14_, t_30_ and t_360_). After each storage time, samples were removed from the freezers and immediately freeze-dried, under the same conditions, to prevent thaw-induced enzymatic and oxidative reactions and standardize results on a dry-matter basis. Thus, freeze-dried samples were analyzed for single and total phenolic compounds and bitter acids, total phenolic content (TPC), ferric reducing antioxidant power (FRAP) and ABTS^•+^ radical-scavenging activity, as detailed in the following sections.

It is noteworthy that freezing and frozen storage temperatures (−20, −30 and −40 °C) were selected based on the glass transition temperature of the maximally freeze-concentrated matrix (T_g_’) of fresh hop cones in order to obtain conditions above and around the T_g_’. Lower temperatures were not investigated since in frozen systems, reaction rates are not only governed by the Arrhenius-type temperature dependence but are also strongly constrained by molecular mobility in the unfrozen phase. When storage is conducted at temperatures close to or below Tg’, the maximally freeze-concentrated matrix attains a highly viscous, glass-like state in which diffusion becomes the main limiting factor. Under these conditions, further decreasing the temperature (e.g., from −40 °C to −50 °C) does not lead to additional water crystallization, while molecular mobility is already extremely low. As a consequence, additional reductions in temperature would be expected to provide only marginal extra benefits in terms of chemical stability, while substantially increasing energy demand and refrigeration costs.

### 2.4. Chemical Characterization of Frozen Hop Cones

Before chemical characterization, frozen hop cone samples, collected at each storage temperature and time, were freeze-dried for 24 h using a Labogene Scanvac Coolsafe freeze-dryer (Allerød, Denmark) operating at 0.316 hPa by gradually increasing the shelf temperature from −40 °C to 0 °C [1]. Freeze-dried samples were subsequently used for phenolic compound extraction following the method of Santarelli et al. [7], with slight modifications. Extraction was carried out by ultrasonic-assisted extraction (100 W, 50 kHz) for 30 min using a solid-to-solvent ratio of 1:30 (*w*/*v*) and a methanol–water mixture (80:20, *v*/*v*) as the extraction solvent. After extraction, the solutions were filtered through PTFE membrane filters (0.45 μm pore size) before analysis.

### 2.5. Determination of Polyphenols and Bitter Acids Content by HPLC-DAD

The content of single phenols, xanthohumol, and bitter acids was evaluated by HPLC analysis using a chromatographic system (Agilent 1100 series, Agilent, Italy) consisting of a vacuum degasser, a quaternary pump, an autosampler, a thermostated column compartment, and a diode array detector (DAD; Agilent Technologies, Milan, Italy). All analytical data were processed by the data management software system (ChemStation 32.1, Agilent Technologies). In particular, the chromatographic conditions used by Akyuz et al. [18] and Bertelli et al. [19] were combined and slightly modified to identify and quantify all the main hop components with a single run. The separation was performed on a reverse phase Zorbax Eclipse XDB-C18 column 4.6 × 150 mm, 5 μm (Agilent Technologies, Milan, Italy), with a pre-guard column, using a flow rate of 1 mL/min, two solvent systems (A: 2% formic acid in distilled water; B: 80% acetonitrile in methanol) and the following gradient program: 0–2 min, 5% B; 2–8 min, 10% B; 8–11 min, 15% B; 11–13 min, 25% B; 13–17 min, 30% B; 17–30 min, 35% B; 30–50 min, 75% B; 50–70 min, 100% B; 70–75 min, 100% B. The post-running time was set at 10 min. The column temperature was regulated at 30 °C, and the sample injection volume was 20 μL. The UV/DAD acquisitions were carried out in the range of 190–500 nm, and chromatograms were collected at 254, 270, 280, 290, 320, 330, and 370 nm. The injection of each sample was performed in triplicate. Twenty-one phenolic compounds (gallic acid, protocatechuic acid, *p*-hydroxy benzoic acid, syringic acid, vanillic acid, cinnamic acid, *p*-coumaric acid, caffeic acid, chlorogenic acid, ferulic acid, catechin, epicatechin, naringenin, luteolin, quercetin, kaempferol, rutin, daidzein, xanthohumol, isoxanthohumol, 8-prenylnaringenin) and six bitter acids (cohumulone, n-humulone, ad-humulone and colupulone, n-lupulone, ad-lupulone) were chromatographically separated and identified. However, since the ICE-4 reference standard consists of a mixture of α- and β-acids rather than pure individual compounds, calibration was performed based on the certified percentage contents provided by the manufacturer, which report n-humulone + adhumulone (n + adhumulone) and n-lupulone + adlupulone (n + adlupulone) as combined values. A six-point calibration curve based on external standard solutions (0–100 ppm) was used for quantification. Single phenols were expressed as µg g^−1^ of dried hops, and bitter acids were expressed as %*w*/*w*. Analyses were carried out in triplicate.

### 2.6. Total Phenolic Content (TPC) and Antioxidant Capacity (AOC) Determination

Total phenolic content (TPC) was measured using the Folin–Ciocalteu method as reported by Georgé et al. [20]. In brief, 120 μL of appropriately diluted hop extract was combined with 600 μL of Folin–Ciocalteu reagent previously diluted 1:10 with deionized water. The mixture was kept at room temperature in the dark for 2 min; subsequently, 960 μL of Na_2_CO_3_ solution (7.5% *w*/*v*) was added. The mixture was incubated at 50 °C for 5 min, and absorbance was recorded at 760 nm. Quantification was performed using a gallic acid calibration curve (0–100 ppm). Data were expressed as mg GAE/g dry matter (dm), and all measurements were conducted in triplicate.

Antioxidant capacity (AOC) was evaluated by considering both radical-scavenging and ferric-reducing activity by the ABTS^•+^ (2,2′-azinobis(3-ethylbenzothiazoline-6-sulfonic acid)) and FRAP (Ferric-Reducing Antioxidant Power) assays, respectively. The ABTS^•+^ assay was conducted according to Sarabandi et al. [21]. Briefly, 2.45 mM of potassium persulfate was mixed with 7 mM of ABTS^+^ and left to react for 12–16 h at room temperature. Thus, 2970 μL of the obtained ABTS^•+^ working solution was combined with 30 μL of opportunely diluted sample, vortexed for 30 s, and kept in the dark for 7 min before measuring absorbance at 734 nm. Trolox standards were prepared and reacted under the same conditions (30 μL Trolox + 2970 μL ABTS^•+^) to build the calibration curve. Results were reported as Trolox equivalent antioxidant capacity (TEAC, μmol Trolox Eq/g dm).

The FRAP assay was conducted according to Benzie and Strain [22]. The FRAP reagent was freshly prepared by combining TPTZ solution (10 mM in 40 mM HCl), FeCl_3_ (iron(III) chloride, 20 mM) and acetate buffer (300 mM, pH 3.6) in a 1:1:10 ratio. Then, 200 μL of appropriately diluted extract was mixed with 1.3 mL of FRAP reagent and incubated at 37 °C for 30 min. Absorbance was measured at 593 nm. FeSO_4_·7H_2_O (iron(II) sulfate heptahydrate) was used for calibration, and results were expressed as μmol Fe^2+^ Eq/g dm. All antioxidant measurements were performed in triplicate.

All absorbance measurements were performed using a P9 UV/VIS double-beam spectrophotometer (Avantor VWR, Co., Ltd., Shanghai, China).

### 2.7. Peroxidase Enzyme Activity (POD)

POD was assessed at the beginning of storage (t_0_) and after long-term frozen storage (t_360_) with the aim of characterizing the baseline enzymatic status of the hop cones and the overall impact of prolonged frozen storage. Peroxidase extraction and activity were performed following Laika et al. [23], with minor adjustments. Freeze-dried hop material (0.5 g) was mixed with 0.1 M KH_2_PO_4_ buffer (pH 6.50) at a 1:2 (*w*:*v*) ratio; the buffer contained 0.5 g of PVPP (polyvinylpyrrolidone; Sigma, St. Louis, MO, USA). The suspension was homogenized on ice for 3 min at 13,500 rpm using an Ultra-Turrax homogenizer (yellow line DI25 basic, IKA-Werke, Staufen, Germany). The homogenate was centrifuged for 10 min at 5000 rpm at 4 °C, after which the supernatant was filtered and used for analysis. POD was determined spectrophotometrically in 10 mm path-length glass cuvettes by combining 25 μL of hop extract with 1450 μL of ABTS solution (20 mmol/L) and 50 μL of H_2_O_2_ (hydrogen peroxide, 0.3% *v*/*v*) as oxidant. The reaction was carried out at 25 °C, and enzyme activity was monitored as the increase in absorbance at 405 nm using a P9 UV/VIS double-beam spectrophotometer (Avantor VWR, Co., Ltd., Shanghai, China). Activity was expressed as the initial reaction rate, calculated from the slope (ΔA/min) of the linear portion of the absorbance–time curve.

### 2.8. Confocal Laser Scanning Microscopy (CLSM)

External and internal single leaves of frozen-stored hop cones were analyzed directly under a glass coverslip using a Nikon A1-R laser scanning confocal imaging system (Nikon Corp., Tokyo, Japan) controlled by the Nikon NIS-Elements software (V4.40). Imaging was performed with a Plan Apo λ 20× objective (numerical aperture: 0.75; working distance: 1.0 mm; refractive index: 1.0). The system was operated in galvanometric scanning mode.

Images were acquired in DU4 detection mode using photomultiplier tube (PMT) detectors and CH Series acquisition to reduce the cross-talk. To exploit the natural autofluorescence of hop secondary metabolites, samples were excited at 488 nm and 561 nm. Fluorescence was collected in two detection channels covering approximately 475–575 nm (blue–green channel) and 545–645 nm (yellow–red channel).

A pinhole size of 99 µm was used, and Z-stacks were acquired using a Nikon A1 Ti ZDrive system with a Z-step of 1 µm. All samples were imaged under identical acquisition settings (laser lines, detection channels, pinhole size, Z-step, and objective) to ensure comparability among samples.

To minimize variability due to the thawing and redistribution of soluble compounds, samples were removed from the freezer, immediately mounted under the coverslip, and imaged following a strictly standardized timing protocol for all replicates.

### 2.9. Statistical Analysis

Data at t_0_ were reported as mean and standard deviation and additionally analyzed by one-way ANOVA. Significant differences were calculated by the Tukey (HSD) test at a significance level of *p* < 0.05. Data reported POD for comparisons between two storage times at the same temperature, analyzed by an unpaired Student’s *t*-test (*p* < 0.05).

Principal Component Analysis (PCA) was conducted to summarize the main sources of variability in the dataset and to visualize sample separation according to freezing temperature and storage duration. Statistics were performed using XLSTAT software (XLSTAT 2021, Addinsoft, Paris, France).

All the data collected after freezing and frozen storage were additionally processed by multivariate ANOVA to highlight the single and combined effects of the freezing temperature (T) and frozen storage time (ST) on chemical properties of hop cones by using STATISTICA for Windows (version 13.3, StatSoftTM, Tulsa, OK, USA) software.

## 3. Results and Discussion

### 3.1. Thermal Properties

**Figure 1:**
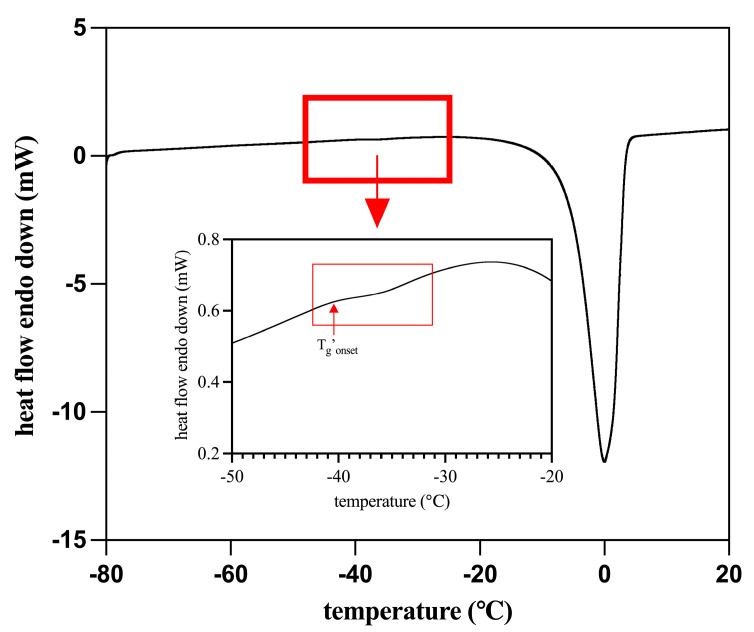
Thermogram of the fresh hop cone indicating the glass transition temperature of the maximally freeze-concentrated phase (T_g_’).

To evaluate the thermal properties of hop cones and select the temperatures for freezing and frozen storage, differential scanning calorimetry (DSC) analysis was performed. The resulting thermogram is shown in Figure 1, including the glass transition temperature of the maximally freeze-concentrated phase (T_g_’). As indicated, the onset of the glass transition of the maximally freeze-concentrated phase was −40.03 ± 0.2 °C (T_g_’_onset_). The freezable water content of the cones, calculated from the area under the melting peak (enthalpy), was determined as 46.47%, which corresponds to the fraction of water capable of crystallizing and transforming into ice during freezing [17].

### 3.2. Enzymatic Activity

To better interpret the results on secondary metabolites and antioxidant activity, peroxidase (POD) activity was determined immediately after freezing (t_0_) and after 360 days of frozen storage (t_360_) (Table 1). Hop cones, in fact, due to the high thermosensitivity of secondary metabolites such as bitter acids, cannot be subjected to an enzymatic stabilization process such as blanching before freezing, and consequently, peroxidase, which is particularly thermostable among the oxidases, may promote oxidative reactions during freezing and frozen storage [24].

At t_0_, cones frozen at −40 °C showed significantly higher POD than those frozen at −30 °C and −20 °C (*p* < 0.05), while no significant difference was observed between −30 °C and −20 °C.

At t_360_, POD remained statistically unchanged in samples stored at −40 °C (*p* > 0.05), whereas a significant increase was observed at −30 °C and −20 °C (*p* < 0.05). By comparing the samples at t_360_, it can be noted that samples stored at −40 °C and −30 °C showed no differences, and both were higher than those stored at −20 °C (*p* < 0.05).

The stability of POD observed at −40 °C is possibly due to rapid freezing, which limits ice-crystal-induced microstructural damage, and storage conditions close to T_g_’, where the highly viscous unfrozen phase reduces molecular mobility. This kinetic limitation hinders not only conformational changes leading to unfolding/aggregation phenomena, but also other degradation pathways such as oxidative modifications, reactions with phenolic-derived quinones [25], and slow chemical changes promoted by cryoconcentration (e.g., pH/ionic-strength shifts), ultimately preserving enzyme activity during frozen storage. Conversely, storage at −30 °C and −20 °C (above T_g_’) may permit higher molecular mobility in the unfrozen phase, promoting ice recrystallization and progressive loss of cellular compartmentalization. These microstructural changes can increase POD extractability/solubilization (including apoplastic or wall-associated isoenzymes) and modify matrix–enzyme interactions, leading to a higher apparent POD in the enzymatic extracts. At −20 °C, increased mobility may also accelerate oxidative/chemical inactivation processes, which could explain why POD at t_360_ remains lower than those at −30 °C and −40 °C despite the overall increase over time.

### 3.3. Effect of Freezing Temperature (t_0_)

**Figure 2:**
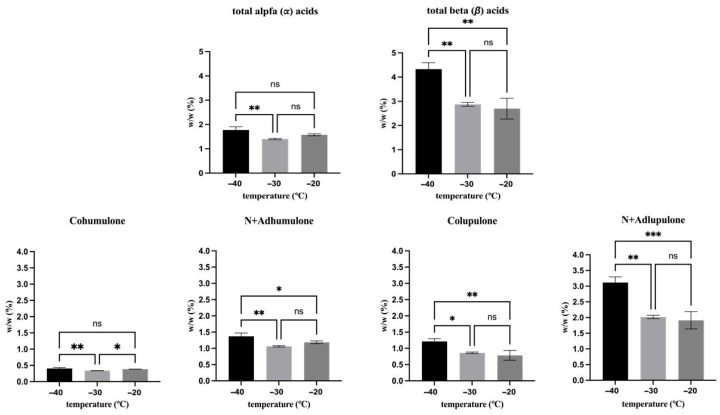
Effect of freezing temperature on the singular and total bitter acids concentration. Asterisks indicate statistically significant differences between treatments: * *p* < 0.05, ** *p* < 0.01, and *** *p* < 0.001, ns, not significant.

**Figure 3:**
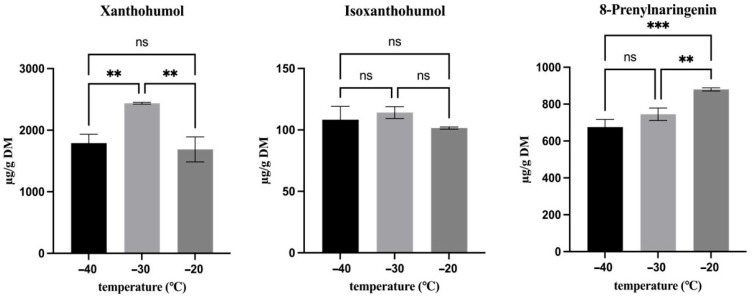
Effect of freezing temperature on the prenylflavonoid concentration. Asterisks indicate statistically significant differences between treatments: ** *p* < 0.01, and *** *p* < 0.001. ns, not significant.

**Figure 4:**
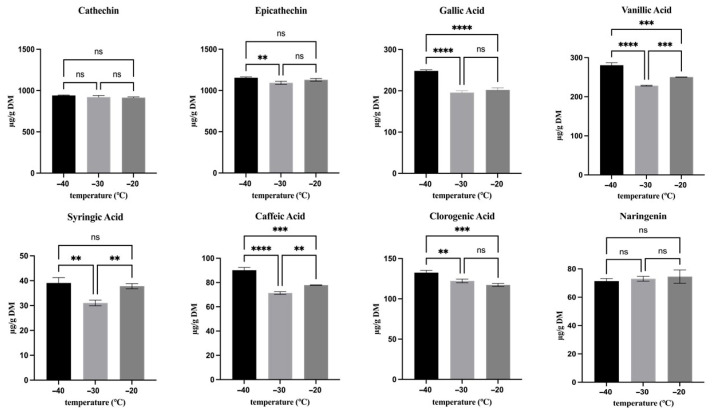
Effect of freezing temperature on the hop phenolic compounds concentration. Asterisks indicate statistically significant differences between treatments: ** *p* < 0.01, and *** *p* < 0.001, **** *p* < 0.0001. ns, not significant.

In order to assess the effect of different freezing temperatures on the hop secondary metabolites derived from lupulin glands (bitter acids and prenylflavonoids) as well as those from bracts and bracteoles (flavon-3-ols, flavanones, benzoic and cinnamic acids and their derivatives), their contents were analyzed immediately after freezing, and the results are presented in Figure 2, Figure 3 and Figure 4.

Concerning bitter acids (Figure 2), hop cones frozen at −40 °C exhibited the highest contents, with concentrations of α-acids and β-acids of 1.77% *w*/*w* and 4.33% *w*/*w*, respectively. Specifically, regarding α-acids, hop cones frozen at −40 °C showed a significantly higher total α-acid content than those frozen at −30 °C (*p* < 0.01), mainly due to the higher levels of cohumulone and n + adhumulone. In contrast, when compared with cones frozen at −20 °C, significantly higher values were observed only for n + adhumulone (*p* < 0.05). Overall, samples frozen at −30 °C and −20 °C did not differ in total or individual α-acid contents (*p* > 0.05).

A clearer temperature effect was observed for β-acids. Samples frozen at −40 °C exhibited significantly higher total β-acid content than both −30 °C and −20 °C (*p* < 0.01), consistent with higher concentrations of colupulone and n + adlupulone. No significant differences were detected between −30 °C and −20 °C for total β-acids or for individual β-acid components (*p* > 0.05).

Regarding prenylflavonoids (Figure 3), a temperature-dependent effect was observed. In particular, the highest concentration of xanthohumol (average 2437 µg g^−1^ dm) was detected in samples frozen at −30 °C, whereas 8-prenylnaringenin showed its maximum content (average 745 µg g^−1^ dm) in those frozen at −20 °C. The concentration of isoxanthohumol did not differ significantly among the differently frozen samples (*p* > 0.05) and highlighted an overall average of 108 µg g^−1^ dm.

In order to discuss the effect of freezing and freezing temperatures on these specific classes of compounds, their location in hop cones should be considered. In fact, they are stored in the lupulin glands, which are not expected to freeze due to their high content of hydrophobic terpenophenolics (resin and oils) [26]. However, the surrounding hydrophobic cuticle, which forms a rigid and non-cellular capsule-like structure around the glands, can be mechanically damaged by the formation of ice crystals during freezing [15]. This could favor the leaching of bitter acids and prenylflavonoids from the glands, thus exposing them to oxidative and enzymatic reactions. On this premise, the lower α- and β-acid contents observed in hop cones frozen at −20 °C and −30 °C compared to those frozen at −40 °C are likely due to the lower freezing rate and higher molecular mobility at these freezing conditions, which allow enzymatic and oxidative reactions to proceed. In fact, under slower freezing conditions, enzymes such as POD and oxygen can more easily interact with their substrates and facilitate the reactions, promoting partial degradation of these compounds [27]. In contrast, rapid freezing at −40 °C more effectively suppresses molecular mobility and reaction kinetics, thereby limiting enzymatic and oxidative processes and resulting in a superior preservation of bitter acids. Although prenylflavonoids are co-localized with bitter acids in lupulin glands, they showed different responses to freezing temperature. Indeed, xanthohumol, isoxanthohumol, and 8-prenylnaringenin possess distinct chemical structures, polarity, and reactivity compared with bitter acids, which influence their stability and extractability under freezing conditions [28,29]. In contrast to α- and β-acids, the highest concentration of prenylflavonoids was not observed at −40 °C, but at −20 °C and −30 °C, and this is possibly due to their enhanced release from lupulin glands. The chemical structure of prenylflavonoids, in fact, offers them slower oxidative transformation pathways compared with bitter acids, which are prone to rapid autoxidation [30]. Moreover, freezing hop cones at −40 °C, as previously discussed, can likely preserve gland integrity more effectively than at −20 °C and −30 °C, preserving bitter acids while limiting the liberation of prenylflavonoids during extraction. Thus, the highest concentration of xanthohumol observed at −30 °C, despite the lower peroxidase activity observed in this sample after freezing, could be due to the combined effect provided by structural damage of lupulin glands that facilitated compound release and the reduced molecular mobility that hindered degradation reactions. On the other hand, the higher concentration of 8-prenylnaringenin observed at −20 °C is likely related to its distinct flavanone structure and higher polarity, which favor its extractability. Unlike chalcones such as xanthohumol, 8-prenylnaringenin lacks an α, β-unsaturated carbonyl system and is therefore less susceptible to rapid oxidative reactions, allowing it to persist even under conditions of increased molecular mobility [28]. Isoxanthohumol showed no significant dependence on freezing temperature, likely due to its relatively stable flavanone structure and limited sensitivity to freezing-induced changes in extractability within the investigated temperature range.

As regards the flavon-3-ols (Figure 4), catechin content (925 µg g^−1^ dm on average) was not significantly (*p* > 0.05) influenced by freezing temperature, whereas epicatechin content (1155 µg g^−1^ dm on average) had slightly higher results at −40 °C compared with the other freezing temperatures.

Among the flavanones (Figure 4), only naringenin was detected, and as observed for catechin, its concentration (73 µg g^−1^ dm on average) was not significantly (*p* > 0.05) affected by the freezing temperature.

Regarding benzoic acid and its derivatives (Figure 4), overall, samples frozen at −40 °C exhibited a significantly (*p* < 0.01) higher concentration of gallic (248 µg g^−1^ dm), syringic (39 µg g^−1^ dm), and vanillic acids (280 µg g^−1^ dm) compared with those frozen at other temperatures. Syringic and vanillic acids were present at higher contents in samples frozen at −20 °C compared with −30 °C, while gallic acid showed no significant (*p* > 0.05) difference between these temperatures.

Regarding the cinnamic acid and its derivatives (Figure 4), only caffeic and chlorogenic acids were identified. Both compounds exhibited higher concentrations in samples frozen at −40 °C (90 µg g^−1^ dm and 132 µg g^−1^ dm, respectively) than in those frozen at −30 °C or −20 °C, as observed for benzoic acid and its derivatives.

Overall, as observed for bitter acids, also the other phenolic compounds showed their highest content after freezing at −40 °C compared to the other freezing temperatures. These results can be ascribed to faster freezing, which limits ice crystal growth and tissue damage, together with reduced molecular mobility. In hop cones, during freezing, both intracellular (including vacuolar) and extracellular water crystallize, and the resulting ice formation can rupture cellular membranes. This damage, in turn, leads to leakage of phenolic compounds contained within bracts and bracteoles from the vacuoles into the cytosol, where they can come into contact with peroxidase and other oxidative enzymes, potentially promoting their degradation. Thus, freezing hop cones at −40 °C minimized structural damage by forming smaller ice crystals, better preserved vacuolar integrity, and hindered these phenomena. Furthermore, −40 °C is near T_g_’_onset_ (see Figure 1), where molecular mobility is strongly reduced, thus all diffusion-limited reactions, including those promoted by peroxidase activity and oxidative degradation, were possibly hindered, resulting in the preservation of phenolic compounds. Consistent with these results, other studies reported that a quick-freezing process and higher freezing rates contributed significantly to the preservation of bioactive compounds in berry fruit and strawberries [31,32]. However, the effect of freezing temperature on phenolic compounds has been reported with varying results in the literature across the different plant matrices [11,33]. Indeed, Korus and Lisiewska [34] reported no significant differences in caffeic acids and other phenolics (especially flavanols) between kale samples frozen at −30 °C and −20 °C, and, also, Polinati et al. [35] observed similar results in apple and orange samples frozen at −18 °C and −70 °C.

**Figure 5:**
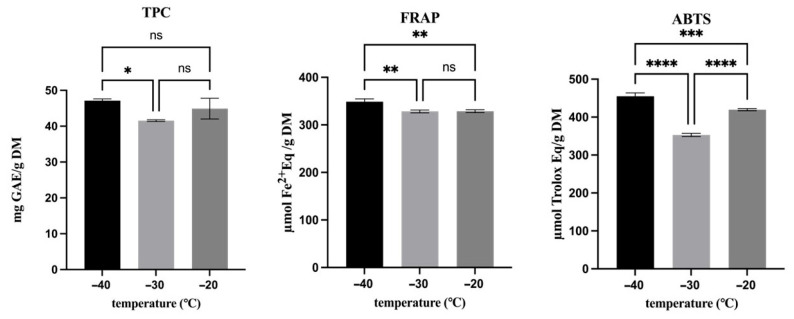
Effect of freezing temperature on the TPC, FRAP and ABTS concentration. Asterisks indicate statistically significant differences between treatments: * *p* < 0.05, ** *p* < 0.01, *** *p* < 0.001 and **** *p* < 0.0001; ns, no significant.

Besides individual phenolic compounds, the effect of the freezing temperature on the TPC and antioxidant activity was also analyzed (Figure 5). Overall, freezing around the T_g_’ exerted a protective effect on TPC and antioxidant properties. Samples frozen at −40 °C exhibited the highest TPC and antioxidant activity measured by both the FRAP and ABTS assays, confirming that rapid freezing at temperatures close to T_g_’ limits molecular mobility and suppresses diffusion-controlled degradative reactions and peroxidase activity. Between −30 °C and −20 °C, no significant differences were observed for TPC or FRAP, indicating that these parameters were unaffected by moderate variations in freezing temperature, once above T_g_’. In contrast, the ABTS assay also revealed a significant difference between −30 °C and −20 °C, with higher values measured in samples frozen at −20 °C, suggesting a higher sensitivity of radical-scavenging capacity to changes in the relative contribution or accessibility of specific antioxidants.

**Figure 6:**
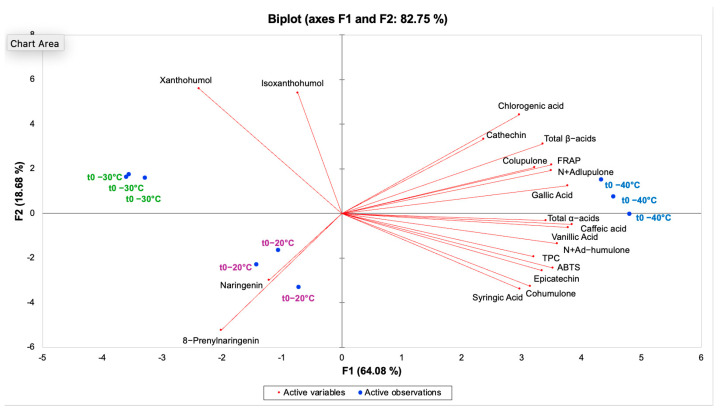
PCA biplot (scores and loadings) of hop cones immediately after freezing (t_0_) at −20, −30 and −40 °C, based on secondary metabolites and antioxidant indices.

To obtain an overview of the overall compositional differences induced by freezing temperature at t_0_, PCA was applied to the dataset of secondary metabolites and antioxidant indices. The biplot reported in Figure 6 shows the first two principal components (PC1 and PC2), which explain, respectively, 64.08% and 18.68% of the variance, accounting for a cumulative 82.75% of the total variance.

PC1 primarily discriminated samples frozen at −40 °C from those frozen at −30 and −20 °C, with the samples frozen at −40 °C being associated with higher bitter acids (total α- and β-acids), phenolic acids and antioxidant indices (TPC, FRAP and ABTS), in agreement with univariate comparisons. PC2 further contributed to the separation between −30 and −20 °C, mainly reflecting differences in the content of xanthohumol/isoxanthohumol and naringenin/8-prenylnaringenin. The biplot also highlighted covariance structures among variables, with antioxidant indices clustering with phenolic compounds and bitter acids, whereas prenylflavonoids loaded in distinct directions, indicating a different temperature-dependent behavior. The PCA patterns observed at −30 and −20 °C (freezing above Tg’) are consistent with a higher molecular mobility in the unfrozen phase, which may facilitate diffusion-limited reactions and, potentially, enzyme-mediated oxidation.

### 3.4. Effect of Freezing and Frozen Storage at Different Temperatures

The changes in individual and total bitter acids during frozen storage at different temperatures are reported in Table 2.

Concerning α-acids, all the samples showed, in general, an increase in these compounds over time at all temperatures, the extent of which was time- and temperature-dependent. After 360 days, in particular, samples stored at −30 °C exhibited a significantly higher cohumulone content (*p* < 0.05) compared with those stored at the other temperatures, whereas for n + adhumulone or for total α-acid content no significant differences (*p* > 0.05) were observed.

Regarding β-acids, the response to storage was even more time- and temperature-dependent than for α-acid (Table 2). Specifically, for both colupulone and n + adlupulone, the lowest variability over time was detected in samples stored at −40 °C. Comparing all the samples at 360 days, those stored at −30 °C showed the highest content (*p* < 0.05) of colupulone, whereas samples stored at −40 and −20 °C showed no significant differences (*p* > 0.05) among each other. For n + adlupulone, the highest values were highlighted in samples stored at −30 °C and −40 °C (*p* > 0.05). As a consequence, compared with t_0_, total β-acids remained essentially stable at −40 °C and −20 °C, whereas an increase of approximately +58% was observed at −30 °C.

Prenylflavonoids showed a compound-specific response (Table 2). Xanthohumol decreased significantly within the first 30 days at all storage temperatures; however, after 360 days, samples stored at −30 °C and −20 °C showed comparable contents (*p* > 0.05) with respect to t_0,_ whereas the sample stored at −40 °C presented a significant increase (about +31%, *p* < 0.05). In contrast, both isoxanthohumol and 8-prenylnaringenin decreased significantly at all storage temperatures (*p* < 0.05), with pronounced losses already evident within the first month of storage. At the end of the storage, the highest isoxanthohumol loss was observed for samples stored at −40 °C, while for 8-prenylnaringenin, no significant differences (*p* > 0.05) were observed among the differently stored samples.

To the best of the authors’ knowledge, the literature data on the variation in bitter acids and prenylflavonoids in hop cones over long-term frozen storage are currently lacking, which hinders the comparison and confirmation of the obtained findings.

Concerning prenylflavonoids, since their interconversion [36] is expected to be minimal at subzero temperatures, the observed decreases are more plausibly attributable to oxidative degradation processes during storage rather than to thermal transformation. In addition to compositional changes, the apparent increases observed for lupulin-associated metabolites (bitter acids and xanthohumol) may also reflect temperature-dependent variations in extractability. These compounds, in fact, are largely concentrated in the resinous fraction within lupulin glands, and freezing/storage may alter gland morphology and the integrity of the glandular envelope, thereby affecting solvent accessibility and mass transfer during extraction.

Finally, the increases observed for bitter acids and xanthohumol might also be partially related to analytical artifacts, such as potential co-elution of additional homologues formed during storage (e.g., products arising from polymerization and/or oxidation of α- and β-acids) [37], which may not have been adequately resolved under the chromatographic conditions employed [38]. Further investigations using complementary techniques (e.g., HPLC–MS/MS or Orbitrap-based HRMS) are therefore needed to identify and quantify additional homologues and/or oxidation products that may have influenced the analytical results.

Concerning phenolic compounds, flavan-3-ols decreased significantly during storage (Table 2) in agreement with what has been observed by other authors in other plant matrices after long-term storage at approximately −20 °C [39,40,41]. In particular, after 360 days of storage, catechin and epicatechin showed a higher decrease at −20 °C compared to those stored at −40 and −30 °C (*p* < 0.05). The reduction was consistently greater for epicatechin than for catechin at all investigated temperatures. This behavior is consistent with the generally higher oxidative susceptibility of epicatechin compared with catechin, as epicatechin exhibits a lower redox potential and higher susceptibility to oxidation, making it a more favorable substrate for oxidative pathways and enzymes [42,43,44,45]. In addition, the more pronounced decrease at −20 °C is coherent with the higher molecular mobility expected at temperatures above T_g_’, which can facilitate diffusion-controlled reactions and residual oxidative activity during storage.

Among the benzoic acid derivatives, both gallic acid and vanillic acid exhibited a decreasing trend over 360 days of storage across all temperatures. At t_360,_ gallic acid showed the greatest loss in samples stored at −20 °C and −30 °C (≈ 95% on average), while the highest concentration (28.21 µg g^−1^) and retention was observed in samples stored at −40 °C. For the vanillic acid, at t_360_, the greatest loss was observed at −40 °C (−66.74%). Concerning syringic acid, it showed a different pattern; irrespective of the storage temperature, its concentration decreased during the first 30 days of storage but drastically increased by the end of the storage time. Specifically, at t_360,_ although the highest concentration (69.08 µg g^−1^) was observed at −40 °C, with respect to t_0,_ the highest variation for this phenolic acid occurred in samples stored at −30 °C (+115%).

Among flavanones, only naringenin was detected. During storage, its concentration decreased at all temperatures, mostly in the first month of storage. After t_30_, naringenin levels remained unchanged up to t_360_ in samples stored at −20 °C and −30 °C (*p* > 0.05), whereas a further significant decrease was observed at −40 °C (*p* < 0.05). In particular, at t_360_ the largest overall loss occurred at −40 °C and −20 °C (≈ 52% on average), while the highest concentration (47.77 µg g^−1^) and lowest loss (35%) was measured in samples stored at −30 °C.

Among the cinnamic acid derivatives, only caffeic acid and chlorogenic acid were detected, and both showed an increasing trend after one month of storage at all the investigated temperatures. At the end of the storage, the highest positive variation and concentration (112 µg g^−1^) of caffeic acid was observed at −30 °C, followed by −20 °C and −40 °C. Chlorogenic acid displayed a similar behavior, with the highest concentration at t_360_ measured in samples stored at −30 °C (165 µg g^−1^), followed by −40 °C and −20 °C.

In order to interpret the behavior of individual phenolic compounds during frozen storage, their structural form and cellular localization should be considered. Phenolic acids occur as soluble free molecules as well as esterified/glycosylated and insoluble-bound forms. Soluble phenolics are mainly compartmentalized in vacuoles, whereas insoluble-bound phenolics (including several hydroxy-benzoic and hydroxy-cinnamic acid derivatives) are embedded in the cell wall matrix and covalently linked to structural polymers; their quantification therefore depends on the extent to which they are released from the matrix during sample processing [46,47]. Freezing and subsequent frozen storage can promote tissue damage and partial decompartmentalization, increasing solvent accessibility and favoring the apparent release of conjugated or wall-associated phenolics [48,49,50]. Within this framework, the late increases observed for syringic acid and for cinnamic derivatives such as caffeic and chlorogenic acids are consistent with a progressive liberation of bound/conjugated pools and with time-dependent changes in extractability. Similar increases in selected benzoic and cinnamic derivatives relative to the initial content have been reported in strawberries after frozen storage, supporting the possibility that long-term frozen storage may shift the measurable balance between “released” and “degraded” pools [51].

The magnitude of these effects is expected to depend on the distance between storage temperature and the onset glass transition temperature (T_g_’), which governs molecular mobility in the freeze-concentrated phase and controls stress-relaxation and recrystallisation kinetics [52]. Storage close to T_g_’ (e.g., −40 °C in the present conditions) is expected to strongly restrict diffusion-limited phenomena, which may limit both oxidative/enzymatic reactions and the progressive release of wall-associated compounds, thereby reducing time-dependent changes in extractability. Conversely, storage well above T_g_’ (e.g., −20 °C) can increase mobility in the freeze-concentrated domains and facilitate diffusion-controlled reactions and matrix reorganization (stress relaxation, water redistribution, and recrystallisation), potentially contributing to larger losses of readily oxidizable soluble phenolics. The intermediate condition (−30 °C) may therefore reflect a combined effect of (i) structural disruption of bract tissues, sufficient to enhance accessibility and release of wall-associated phenolics, and (ii) molecular mobility that is still limited enough to slow down degradation pathways. Consistently, at −30 °C, the decrease in soluble bract-associated compounds such as vanillic acid and naringenin was less pronounced than those measured at other temperatures, while the increases relative to t_0_ were more evident for syringic acid and for caffeic/chlorogenic acids.

Regarding total phenolic content (TPC), values decreased at all storage temperatures (Table 2), in line with the overall behavior observed for individual phenolics. Most of the reduction occurred within the first 7 days, after which TPC remained quite stable. At t_360_, no significant differences were observed in TPC between the investigated temperatures (*p* < 0.05). The rapid early decrease may reflect oxidative and or enzymatic reactions occurring during the initial freezing phase, when partial ice crystallization still allows limited molecular mobility [53]. A similar pattern, with an initial decline followed by stabilization during prolonged frozen storage, has been reported for other phenolic-rich plant matrices, including raspberries and vegetables [54,55].

During storage, antioxidant activity measured by both FRAP and ABTS (TEAC) generally decreased at all storage temperatures (Table 2). It should be noted that ABTS (TEAC) showed its largest decrease within the first month, followed by a partial recovery by the t_360_, whereas FRAP displayed a more variable profile, especially at early storage times. At t_360_, similar to TPC, no significant differences were observed for FRAP nor for ABTS at any investigated temperatures (*p* > 0.05).

Consistent with the present study’s results, Korus and Lisiewska [34] did not report significant differences in the antioxidant value measured with ABTS of kale leaves stored at −20 and −30 °C. On the other hand, Gebczynski [56] and Gebczynski and Lisiewska [57] investigated the antioxidant activity of cauliflower and broccoli stored at −20 °C and −30 °C and reported that storage at −30 °C better preserved antioxidant compounds, resulting in the highest antioxidant activity values.

**Figure 7:**
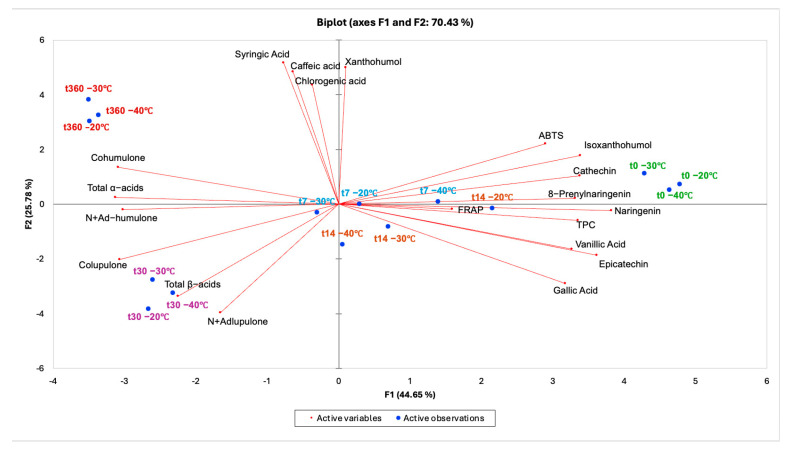
PCA biplot shows the relationship between the chemical composition of hop cones frozen and stored at different temperatures up to 360 days.

Principal component analysis (PCA) was performed on the full dataset (individual and total bitter acids, prenylflavonoids, phenolic compounds, TPC and antioxidant indices) to provide an integrated view of compositional changes induced by frozen storage time and storage temperature (Figure 7).

The first two principal components explained 70.43% of total variance (PC1 = 44.65%, PC2 = 25.78%), indicating an adequate representation of the multivariate structure.

Overall, it can be observed that sample separation was primarily driven by storage time. Along PC1, samples t_0_ clustered at high PC1 scores, in the direction of antioxidant-related variables and phenolic markers (TPC and ABTS, together with catechin/epicatechin, naringenin and 8-prenylnaringenin), whereas t_30_ and t_360_ days shifted to negative PC1 values. This pattern indicates a time-dependent change from a profile characterized by extractable phenolics and antioxidant activity (positive PC1) toward a profile more strongly associated with bitter-acid-related variables (negative PC1), reflecting the overall decrease in several phenolics and antioxidant indices during storage and with the apparent increase in bitter acids observed at long times.

PC2 further discriminated the samples subjected to frozen storage, separating t_30_ samples (negative PC2) from t_360_ samples (positive PC2). The negative PC2 region was mainly associated with total β-acids, colupulone and n + adlupulone, in agreement with the observed maximum of β-acids at t_30_. In contrast, the positive PC2 region was associated with xanthohumol and phenolic acids (notably syringic, caffeic and chlorogenic acids), consistent with their higher contribution at t_360_. Therefore, the PCA suggests a temporal “trajectory” from t_0_ (high PC1) to an intermediate region (t_7_–t_14_), and finally to t_360_ (negative PC1/positive PC2), highlighting that early stages were mainly associated with phenolic/antioxidant variables, whereas t_30_ and t_360_ were primarily associated with β-acids and with xanthohumol/phenolic acids, respectively. Regarding temperature, samples stored at −20, −30 and −40 °C generally clustered close to each other within the same sampling time, indicating that storage time explained most of the variability captured by the principal components, whereas temperature contributed less to the overall separation. However, temperature-dependent differences were more visible at intermediate times (t_7_–t_14_), whereas at t_30_ and t_360_ the samples converged to a more similar positioning in the PCA space.

To statistically support the PCA observations, a multifactorial ANOVA (MANOVA) was conducted using storage temperature (T) and storage time (ST) as factors. Results highlighted a significant effect for all the individual and combined factors under investigation, except for isoxanthohumol and FRAP, the results of which were not affected by T. As observed for PCA, MANOVA showed that secondary metabolites of hop samples were mostly influenced by ST rather than by T (Supplementary Table S1).

### 3.5. Confocal Microscope Analysis

**Figure 8:**
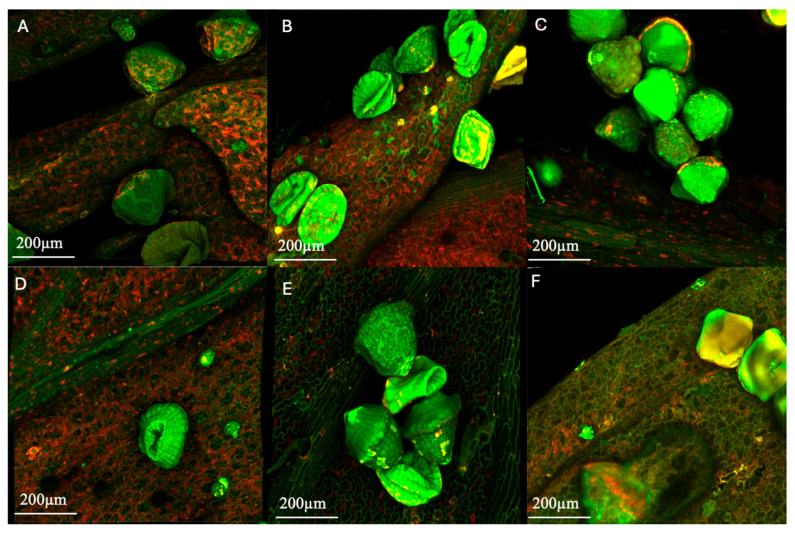
CLSM micrographs of external (**A**–**C**) and internal hop leaves (**D**–**F**) stored for 360 days at −40, −30, and −20 °C, respectively.

To support the interpretation of hop secondary-metabolite changes after 360 days of frozen storage, confocal micrographs of external (Figure 8A–C and Figure S4–S6) and internal (Figure 8D–F and Figure S4–S6) hop cone bracts were acquired by exploiting endogenous autofluorescence (Ex 488 and 561 nm). Images were acquired in DU4 detection mode. Fluorescence was collected in two partially overlapping detection channels, i.e., 475–575 nm (“blue–green channel”) and 545–645 nm (“red–orange channel”). Thus, the images shown are a merge of the two channels (pseudo-colors). Specifically, green pixels are mostly due to the 488/475–575 signal, red–orange areas are due to the 561/545–645 signal, and yellow regions indicate pixels where both channels contribute (cross-talk).

Under these acquisition conditions, the detected signals must be interpreted as composite autofluorescence arising from several endogenous fluorophores, and the selected wavelength intervals must be intended as instrumental detection bands, rather than as intrinsic emission ranges of single compounds. Specifically, in plant tissues, blue–green autofluorescence under visible excitation mainly derives at the cell wall level from lignin and bound phenolic compounds, and at the intracellular level from polyphenols and other cell components [58,59]; conversely, longer-wavelength emissions are commonly associated with flavonoids and related phenolic compounds.

Importantly, chlorophyll fluorescence typically shows dominant emission peaks at ~685 nm with a shoulder extending to 700–750 nm. However, in the present study, with an upper detection limit of 645 nm, chlorophyll emission is largely excluded from the collected signals and therefore cannot be considered the main contributor to the “red” channel.

In hops, as indicated by do Nascimento et al. [60], bitter acids emit mainly between 450 and 580 nm (maximum around 510 nm), while xanthohumol, which can be found both in lupulin glands and hop bracts [61], emits between approximately 520 and 650 nm (maximum around 550 nm). Lupulin glands, therefore, typically show composite emission profiles with maxima at about 505–525 nm plus a ~550 nm component, and a chlorophyll-related band above 650 nm.

Accordingly, the 475–575 nm channel is consistent with fluorescence from bitter acids and other phenolic/flavonoid-type fluorophores, whereas the 545–645 nm channel mainly captures the yellow–orange emission component of hop fluorophores (notably xanthohumol around ~550 nm, plus the long-wavelength tail of the 450–580 nm band). Furthermore, hop essential oils, which are mainly located in the lupulin glands, are weakly or non-fluorescent and therefore are expected to contribute only marginally to hop autofluorescence [62].

On this basis, observing the CLSM micrographs of samples t_360_ stored at −40 °C (Figure 8A,D and Figure S4), it can be noted that cells presented cell walls that were well defined by green autofluorescence, likely due to contributions from phenolic fluorophores such as flavonoids and cinnamic acids [63], and intracellular components highlighting intense red fluorescence. As concerns lupulin glands, they showed some alterations from their characteristic mushroom-like morphology [15] and highlighted green autofluorescence, possibly due to the bitter-acid-related emission band. Localized yellow/orange pixels indicate an increased contribution of the 545–645 nm detection channel, which is compatible with xanthohumol emission and/or spectral overlap between the two detection bands in the 545–575 nm region.

Samples stored at −30 °C (Figure 8B,E and Figure S5), compared to those stored at −40 °C, exhibited cell walls with a similar shape but with slightly higher localized green fluorescence, while lupulin glands presented similar morphology but a stronger green autofluorescence.

Finally, samples stored at −20 °C (Figure 8C and Figure S6) exhibited cell walls that were altered in shape and much less defined, with a more pronounced and distributed red fluorescence and some artifacts due to surrounding visible water layers, indicative of higher water released by the tissue during sample thawing. Such results suggest more extensive tissue disruption and lixiviation of cell components on the cell surface. Lupulin glands showed surrounding visible water layers and oversaturation (both green and yellow) phenomena, possibly indicating a higher local concentration and/or exposure of strongly autofluorescent secondary metabolites, consistent with partial gland rupture and leakage of lupulin-associated compounds onto the surrounding tissue.

In particular, the intense 475–575 nm signal (green autofluorescence) is compatible with an enrichment of bitter acids and other phenolic-type fluorophores, while the increased yellow/orange contribution may reflect a higher relative contribution of the 545–645 nm channel (e.g., prenylflavonoids such as xanthohumol) and/or enhanced cross-talk due to very high signal levels. The concomitant water/exudate film could further amplify the recorded intensity by reducing scattering and acting as a refractive-index-matching layer, thereby increasing the fraction of emitted photons reaching the detector and favoring signal saturation/clipping.

Overall, these features are consistent with more extensive freeze-induced structural damage at −20 °C, leading to discontinuities in the gland envelope and cell walls and promoting the leaching/redistribution of fluorescent compounds at the tissue surface. Since saturated pixels do not retain quantitative intensity information, these observations should be interpreted qualitatively as indicators of increased heterogeneity and surface deposition of endogenous fluorophores rather than as quantitative differences in concentration.

## 4. Conclusions

This study provides a comprehensive evaluation of the effects of different freezing and frozen storage temperatures on the stability of hop secondary metabolites by comparing their chemical profile, antioxidant activity, and conducting a microstructural analysis. A physicochemical perspective is adopted to interpret temperature-dependent changes in hop quality after freezing and during long-term frozen storage by selecting freezing and storage temperatures in relation to the glass transition temperature of the maximally freeze-concentrated matrix (T_g_’_onset_ ≈ −40 °C).

Freezing at −40 °C was most effective in preserving bitter acids, total phenolics, and antioxidant activity, consistent with highly limited molecular mobility and oxidative reactions near the Tg’. In contrast, xanthohumol showed its highest concentration freezing at −30 °C.

Regarding frozen storage, a combined evaluation of storage time and temperature showed that time predominantly drove the evolution of hop bioactives and antioxidant properties over 360 days, whereas temperature mainly modulated the magnitude of these time-dependent changes. Most of the phenolic compounds decreased over storage, while bitter acids and xanthohumol showed variable trends with apparent increases possibly due to improved extractability driven by microstructural changes, and/or the development of additional homologues and/or oxidation products. These aspects need to be further investigated using complementary analytical techniques (e.g., HPLC–MS/MS or Orbitrap-based HRMS). CLSM analysis proved to be a valuable tool for highlighting the effects of different temperatures on hop cone microstructure after long-term frozen storage.

Overall, these findings demonstrate that the selection of freezing temperatures and storage time strongly influence the composition of hop cones, highlighting other possible uses as a source of high-value bioactive compounds for pharmaceutical, nutraceutical, and cosmetic applications beyond the brewing industry.

## Figures and Tables

**Table 1:** Peroxidase (POD) activity after freezing (t_0_) and frozen storage (t_360_) at different temperatures.

		Enzymatic Activity U/g dm
Temperature (°C)	Time (Days)	
−40	0	59,490 ± 1540 ^aA^
	360	59,212 ± 1181 ^aA^
−30	0	30,348 ± 1352 ^bB^
	360	64,723 ± 1947 ^aA^
−20	0	34,389 ± 945 ^bB^
	360	50,695 ± 3108 ^bA^

Lowercase letters indicate statistically significant differences among samples stored at different temperatures at equal storage time (one-way ANOVA followed by Tukey’s post hoc test), whereas capital letters indicate statistically significant differences between samples stored at the same temperature for different storage times (unpaired Student’s *t*-test) (*p* < 0.05).

**Table 2:** Changes in bitter acids, prenylflavonoids, polyphenols, total phenolic content and antioxidant capacity during frozen storage at different temperatures.

			**Compound**						
			**Bitter Acids (*w*/*w* %)**						
Temperature (°C)	Time (days)		Cohumulone	N + adhumulone	total α-acids	Colupulone	N + Adlupulone	total β-acids	
								
−40	0		0.41 ± 0.02 ^dA^	1.37 ± 0.10 ^cA^	1.77 ± 0.13 ^cA^	1.21 ± 0.08 ^bA^	3.11 ± 0.18 ^bA^	4.33 ± 0.26 ^bA^	
							
	7		1.15 ± 0.09 ^bB^	3.41 ± 0.25 ^aB^	4.07 ± 0.92 ^abB^	1.61 ± 0.12 ^bB^	2.71 ± 0.19 ^bB^	4.32 ± 0.31 ^bB^	
			(+183.85%)	(+148.84%)	(+129.39%)	(+32.88%)	(−12.98%)	(−0.13%)	
	14		0.72 ± 0.17 ^cA^	2.36 ± 0.56 ^bA^	3.08 ± 0.73 ^bcA^	1.64 ± 0.49 ^bA^	3.21 ± 0.81 ^bA^	4.85 ± 1.31 ^bA^	
			(+76.24%)	(+72.81%)	(+73.82%)	(+35.44%)	(+2.96%)	(+12.05%)	
	30		0.92 ± 0.15 ^bcB^	3.15 ± 0.51 ^abA^	4.08 ± 0.66 ^abA^	2.43 ± 0.33 ^aA^	4.66 ± 0.66 ^aA^	7.09 ± 0.98 ^aA^	
			(+127.65%)	(+130.22%)	(+129.93%)	(+100.31%)	(+49.48%)	(+63.72%)	
	360		1.46 ± 0.05 ^aB^	3.57 ± 0.14 ^aAB^	5.04 ± 0.18 ^aAB^	1.94 ± 0.08 ^abAB^	2.35 ± 0.10 ^bA^	4.29 ± 0.18 ^bA^	
			(+259.64%)	(+161.00%)	(+183.98%)	(+60.36%)	(−24.58%)	(−0.79%)	
								
−30	0		0.34 ± 0.004 ^cB^	1.06 ± 0.02 ^eB^	1.40 ± 0.02 ^eB^	0.86 ± 0.02 ^bB^	2.02 ± 0.05 ^cB^	2.87 ± 0.07 ^dB^	
							
	7		1.47 ± 0.04 ^aA^	4.99 ± 0.10 ^aA^	6.73 ± 0.15 ^aA^	2.24 ± 0.04 ^aA^	3.73 ± 0.09 ^bA^	5.97 ± 0.13 ^bA^	
			(+414.01%)	(+369.48)	(+380.27)	(+160.78%)	(+85.05%)	(+107.68%)	
	14		0.53 ± 0.03 ^cA^	1.77 ± 0.09 ^dA^	2.30 ± 0.12 ^dA^	0.93 ± 0.04 ^bAB^	2.29 ± 0.02 ^cA^	3.22 ± 0.06 ^dA^	
			(+56.61%)	(+66.63%)	(+64.20%)	(+8.77%)	(+13.49%)	(+12.08%)	
	30		0.94 ± 0.05 ^bAB^	3.22 ± 0.09 ^cA^	4.16 ± 0.24 ^cA^	2.42 ± 0.09 ^aA^	4.58 ± 0.15 ^aA^	7.01 ± 0.25 ^aA^	
			(+176.94%)	(+203.20%)	(+196.83%)	(+181.95%)	(+127.32%)	(+143.64%)	
	360		1.77 ± 0.20 ^aA^	3.97 ± 0.29 ^bA^	5.74 ± 0.50 ^bA^	2.09 ± 0.28 ^aA^	2.43 ± 0.33 ^cA^	4.53 ± 0.61 ^cA^	
			(+421.00%)	(+273.81%)	(+309.47%)	(+143.85%)	(+20.76%)	(+57.54%)	
								
−20	0		0.38 ± 0.002 ^bA^	1.187 ± 0.04 ^cA^	1.57 ± 0.04 ^bAB^	0.78 ± 0.15 ^cB^	1.91 ± 0.28 ^bcB^	2.69 ± 0.43 ^cdB^	
							
	7		1.27 ± 0.20 ^aB^	3.55 ± 0.33 ^abB^	4.82 ± 0.53 ^aB^	1.61 ± 0.23 ^bB^	2.74 ± 0.39 ^bB^	4.35 ± 0.63 ^bB^	
			(+228.95%)	(+198.89%)	(+206.10%)	(+104.86%)	(+43.42%)	(+61.28%)	
	14		0.12 ± 0.001 ^bB^	0.29 ± 0.06 ^dB^	0.41 ± 0.006 ^cB^	0.36 ± 0.05 ^cB^	0.92 ± 0.01 ^dB^	1.28 ± 0.02 ^dB^	
			(−68.93%)	(−75.57%)	(−73.95%)	(−53.96%)	(−51.84%)	(−52.46%)	
	30		1.25 ± 0.16 ^aA^	4.06 ± 0.43 ^aA^	5.35 ± 0.59 ^aA^	2.99 ± 0.36 ^aA^	5.53 ± 0.62 ^aA^	8.57 ± 0.97 ^aA^	
			(+223.87%)	(+242.48%)	(+237.72%)	(+281.41%)	(+188.81%)	(+215.73%)	
	360		1.35 ± 0.001 ^aB^	3.22 ± 0.004 ^bB^	4.58 ± 0.005 ^aB^	1.54 ± 0.005 ^bB^	1.80 ± 0.005 ^cdB^	3.34 ± 0.01 ^bcB^	
			(+250.64%)	(+171.72%)	(+190.94%)	(+96.79%)	(−5.93%)	(+23.94%)	
			**Prenylflavonoids (µg/g)**				**Flavan-3-ols (µg/g)**		
			Xanthohumol	Isoxanthohumol	8-Prenylnaringenin		Catechin	Epicatechin	
							
−40	0		1789.23 ± 144.09 ^bB^	108.38 ± 10.81 ^aA^	675.94 ± 40.44 ^aB^		940.26 ± 5.21 ^aA^	1154.74 ± 9.95 ^aA^	
						
	7		1693.07 ± 23.59 ^bcA^	71.84 ± 16.99 ^bA^	429.46 ± 32.64 ^bB^		797.51 ± 2.61 ^bA^	980.95 ± 26.65 ^bA^	
			(−5.37%)	(−32.79%)	(−36.47%)		(−15.18%)	(−15.05%)	
	14		1402.04 ± 151.87 ^cdA^	15.05 ± 0.11 ^cdB^	186.24 ± 18.94 ^cB^		685.33 ± 63.07 ^cA^	836.48 ± 60.01 ^cAB^	
			(−21.64%)	(−86.12%)	(−72.45%)		(−27.11%)	(−27.56%)	
	30		1236.70 ± 116.73 ^dA^	6.86 ± 1.33 ^dAB^	91.23 ± 7.63 ^dB^		509.55 ± 11.57 ^dB^	776.62 ± 31.62 ^cA^	
			(−30.88%)	(−93.67%)	(−86.50%)		(−45.81%)	(−32.75%)	
	360		2350.44 ± 53.25 ^aAB^	23.51 ± 0.84 ^cB^	47.22 ± 4.34 ^dA^		680.22 ± 15.59 ^cA^	432.13 ± 8.05 ^dB^	
			(+31.37%)	(−78.31%)	(−93.01%)		(−27.66%)	(−62.58%)	
							
−30	0		2437.27 ± 14.59 ^aA^	114.12 ± 4.78 ^aA^	745.46 ± 33.59 ^aB^		920.82 ± 19.51 ^aA^	1093.43 ± 19.12 ^aB^	
						
	7		1756.12 ± 24.75 ^bA^	63.34 ± 1.27 ^bA^	759.47 ± 4.57 ^aA^		719.92 ± 2.04 ^bB^	957.73 ± 16.21 ^bAB^	
			(−27.95)	(−44.50%)	(+1.88%)		(−21.82%)	(−12.41%)	
	14		1488.79 ± 97.87 ^bcA^	24.51 ± 1.03 ^cA^	242.83 ± 6.65 ^bA^		704.33 ± 18.57 ^bA^	750.58 ± 30.12 ^cB^	
			(−38.92%)	(−78.52%)	(−67.43%)		(−23.51%)	(−31.36%)	
	30		1343.07 ± 11.43 ^cA^	4.99 ± 0.45 ^dB^	149.21 ± 24.50 ^cA^		558.99 ± 12.81 ^dA^	740.08 ± 14.89 ^cA^	
			(−44.89%)	(−95.63%)	(−79.99%)		(−39.29%)	(−32.31%)	
	360		2637.23 ± 244.58 ^aA^	27.67 ± 0.23 ^cA^	50.79 ± 2.52 ^dA^		653.88 ± 3.93 ^cA^	475.43 ± 17.23 ^dA^	
			(+8.20%)	(−75.76%)	(−93.19%)		(−28.99%)	(−62.58%)	
							
−20	0		1688.44 ± 203.74 ^abB^	101.54 ± 0.92 ^aA^	879.47 ± 8.37 ^aA^		913.58 ± 10.49 ^aA^	1129.17 ± 16.47 ^aAB^	
						
	7		1684.91 ± 70.42 ^bA^	63.49 ± 0.95 ^bA^	730.54 ± 9.91 ^bA^		639.66 ± 10.46 ^bC^	927.48 ± 8.65 ^bB^	
			(−0.21%)	(−37.47%)	(−16.93%)		(−29.98%)	(−17.86%)	
	14		1696.65 ± 197.78 ^abA^	24.47 ± 1.65 ^dA^	261.12 ± 27.85 ^cA^		578.84 ± 11.29 ^cB^	881.68 ± 20.06 ^bA^	
			(+0.49%)	(−75.90%)	(−70.31%)		(−36.64%)	(−21.92%)	
	30		1027.11 ± 6.42 ^cB^	8.19 ± 0.70 ^eA^	131.62 ± 11.33 ^dAB^		591.79 ± 17.49 ^bcA^	727.47 ± 40.97 ^cA^	
			(−39.17%)	(−91.94%)	(−85.03%)		(−35.22%)	(−35.57%)	
	360		2036.77 ± 0.68 ^aB^	28.02 ± 1.05 ^cA^	44.07 ± 0.48 ^eA^		427.80 ± 37.88 ^dB^	269.22 ± 3.08 ^dC^	
			(+20.63%)	(−72.41%)	(−94.99%)		(−53.17%)	(−76.16%)	
			**Benzoic acids and derivatives (µg/g)**						
			Gallic acid	Protocatechuic acid	Syringic acid		Vanillic acid		
						
−40	0		248.17 ± 2.88 ^aA^	n.d.	39.07 ± 2.15 ^bA^		280.54 ± 6.36 ^aA^		
					
	7		189.16 ± 10.77 ^bcB^	n.d.	38.68 ± 1.59 ^bA^		276.09 ± 9.58 ^aA^		
			(−23.78%)		(−1.02%)		(−1.58%)		
	14		196.37 ± 12.26 ^bA^	n.d.	32.36 ± 0.76 ^cA^		230.08 ± 5.99 ^bA^		
			(−20.88%)		(−17.19%)		(−17.98%)		
	30		170.11 ± 2.39 ^cA^	n.d.	12.01 ± 1.12 ^dB^		163.49 ± 3.55 ^cA^		
			(−31.45%)		(−69.27%)		(−41.72%)		
	360		28.21 ± 0.91 ^dA^	n.d.	69.08 ± 0.19 ^aA^		93.30 ± 0.83 ^dA^		
			(−88.63%)		(+76.80%)		(−66.74%)		
						
−30	0		195.72 ± 4.48 ^aB^	n.d.	31.05 ± 1.13 ^cB^		228.43 ± 1.17 ^bC^		
					
	7		198.29 ± 0.75 ^aB^	n.d.	39.59 ± 2.52 ^bA^		283.79 ± 1.29 ^aA^		
			(+1.31%)		(+27.51%)		(+24.23%)		
	14		149.08 ± 2.12 ^bB^	n.d.	33.20 ± 1.62 ^cA^		198.91 ± 10.14 ^cB^		
			(−23.83%)		(+6.95%)		(−12.93%)		
	30		133.06 ± 1.24 ^cC^	n.d.	16.15 ± 0.36 ^dA^		158.15 ± 1.23 ^dA^		
			(−32.01%)		(−47.99%)		(−30.77%)		
	360		12.32 ± 0.26 ^dB^	n.d.	66.60 ± 0.73 ^aB^		97.24 ± 7.03 ^eA^		
			(−93.70%)		(+114.51%)		(−57.43%)		
						
−20	0		202.12 ± 4.98 ^bB^	n.d.	37.79 ± 1.03 ^bA^		250.28 ± 0.43 ^aB^		
					
	7		216.65 ± 1.33 ^aA^	n.d.	36.17 ± 0.15 ^cA^		258.12 ± 3.99 ^aB^		
			(+7.19%)		(−4.30%)		(+3.13%)		
	14		212.49 ± 3.08 ^aA^	n.d.	32.61 ± 0.40 ^dA^		224.95 ± 5.13 ^bA^		
			(+5.13%)		(−13.72%)		(−10.12%)		
	30		148.03 ± 2.41 ^cB^	n.d.	11.27 ± 0.11 ^eB^		165.56 ± 14.96 ^cA^		
			(−26.76%)		(−70.19%)		(−33.85%)		
	360		9.12 ± 0.85 ^dC^	n.d.	60.99 ± 0.31 ^aC^		92.25 ± 4.57 ^dA^		
			(−95.49%)		(+61.35%)		(−63.14%)		
			**Cinnamic acid and derivatives (µg/g)**						
			Cinnamic acid	*p*-Coumaric acid	Caffeic acid		Chlorogenic acid		Ferulic acid
							
−40	0		n.d.	n.d.	90.12 ± 2.27 ^aA^		132.53 ± 2.82 ^aA^		n.d.
						
	7		n.d.	n.d.	58.91 ± 3.51 ^bcC^		141.11 ± 26.13 ^aA^		n.d.
					(−34.67%)		(+6.47%)		
	14		n.d.	n.d.	67.91 ± 1.761 ^bA^		86.76 ± 0.87 ^bB^		n.d.
					(−24.65%)		(−34.54%)		
	30		n.d.	n.d.	50.39 ± 6.69 ^cA^		97.31 ± 0.514 ^bA^		n.d.
					(−44.09%)		(−26.58%)		
	360		n.d.	n.d.	99.79 ± 1.36 ^aC^		149.47 ± 2.23 ^aB^		n.d.
					(+10.69%)		(+12.78%)		
							
−30	0		n.d	n.d	71.37 ± 1.12 ^bC^		122.21 ± 2.22 ^cB^		n.d
						
	7		n.d.	n.d.	65.28 ± 1.37 ^cB^		141.98 ± 0.83 ^bA^		n.d.
					(−8.53%)		(+16.18%)		
	14		n.d.	n.d.	50.06 ± 0.13 ^dB^		94.98 ± 1.88 ^dA^		n.d.
					(−29.87%)		(−22.28%)		
	30		n.d.	n.d.	51.42 ± 0.51 ^dA^		95.43 ± 0.16 ^dA^		n.d.
					(−27.96%)		(−21.92%)		
	360		n.d.	n.d.	111.89 ± 0.68 ^aA^		164.97 ± 4.26 ^aA^		n.d.
					(+56.77%)		(+34.99%)		
							
−20	0		n.d	n.d	77.88 ± 0.23 ^bB^		117.19 ± 1.81 ^bB^		n.d
						
	7		n.d.	n.d.	75.51 ± 2.731 ^bA^		129.63 ± 0.93 ^aA^		n.d.
					(−3.05%)		(+10.61%)		
	14		n.d.	n.d.	67.66 ± 0.43 ^cA^		86.24 ± 0.05 ^cB^		n.d.
					(−13.13%)		(−26.42%)		
	30		n.d.	n.d.	54.64 ± 1.84 ^dA^		81.84 ± 2.90 ^cB^		n.d.
					(−29.85%)		(−30.17%)		
	360		n.d.	n.d.	105.98 ± 0.04 ^aB^		133.76 ± 0.98 ^aC^		n.d.
					(+36.07%)		(+14.14%)		
			**Flavanone (µg/g)**		**Flavonol (µg/g)**				**Isoflavone (µg/g)**
			Naringenin	Luteolin	Quercetin		Kaempferol	Rutin	Daidzein
					
−40	0		71.37 ± 1.82 ^aA^	n.d.	n.d.		n.d.	n.d.	n.d.
				
	7		62.29 ± 0.83 ^bA^	n.d.	n.d.		n.d.	n.d.	n.d.
			(−12.73%)						
	14		62.47 ± 1.09 ^bAB^	n.d.	n.d.		n.d.	n.d.	n.d.
			(−12.47%)						
	30		50.08 ± 3.44 ^cA^	n.d.	n.d.		n.d.	n.d.	n.d.
			(−29.84%)						
	360		33.19 ± 1.01 ^dB^	n.d.	n.d.		n.d.	n.d.	n.d.
			(−53.50%)						
					
−30	0		73.02 ± 1.71 ^aA^	n.d.	n.d.		n.d.	n.d.	n.d.
				
	7		54.47 ± 2.43 ^bcB^	n.d.	n.d.		n.d.	n.d.	n.d.
			(−25.39%)						
	14		57.45 ± 3.11 ^bB^	n.d.	n.d.		n.d.	n.d.	n.d.
			(−21.33%)						
	30		46.42 ± 0.04 ^dA^	n.d.	n.d.		n.d.	n.d.	n.d.
			(−36.43%)						
	360		47.77 ± 3.95 ^cdA^	n.d.	n.d.		n.d.	n.d.	n.d.
			(−34.58%)						
					
−20	0		74.53 ± 4.72 ^aA^	n.d.	n.d.		n.d.	n.d.	n.d.
				
	7		47.44 ± 2.05 ^bC^	n.d.	n.d.		n.d.	n.d.	n.d.
			(−36.35%)						
	14		69.72 ± 5.16 ^aA^	n.d.	n.d.		n.d.	n.d.	n.d.
			(−6.46%)						
	30		38.43 ± 2.55 ^bcB^	n.d.	n.d.		n.d.	n.d.	n.d.
			(−48.45%)						
	360		37.84 ± 0.39 ^cB^	n.d.	n.d.		n.d.	n.d.	n.d.
			(−49.23%)						
			TPC (mg GAE/g DM)		FRAP (µmol Fe^2+^ Eq/g DM)			TEAC (µmol Trolox Eq/g DM)	
					
−40	0		47.14 ± 0.49 ^aA^		348.89 ± 5.93 ^aA^			454.99 ± 9.01 ^aA^	
				
	7		35.49 ± 0.44 ^bA^		362.67 ± 26.08 ^aA^			361.43 ± 39.95 ^bA^	
			(−24.71%)		(+3.95%)			(−20.56%)	
	14		35.10 ± 0.09 ^bAB^		207.13 ± 0.97 ^cC^			268.02 ± 3.13 ^cA^	
			(−25.53%)		(−40.63%)			(−41.09%)	
	30		32.08 ± 0.48 ^cB^		277.08 ± 2.37 ^bB^			156.09 ± 3.33 ^dC^	
			(−31.94%)		(−20.58%)			(−65.69%)	
	360		29.75 ± 2.11 ^cA^		278.21 ± 26.71 ^bA^			282.88 ± 22.06 ^cA^	
			(−36.89%)		(−20.26%)			(−37.83%)	
					
−30	0		42.55 ± 0.17 ^aB^		328.34 ± 2.90 ^aB^			352.88 ± 4.23 ^aC^	
				
	7		33.67 ± 0.34 ^cB^		349.05 ± 21.20 ^aA^			341.24 ± 2.71 ^aAB^	
			(−18.95%)		(+6.31%)			(−3.30%)	
	14		35.37 ± 0.93 ^bA^		298.42 ± 9.08 ^bA^			233.35 ± 14.36 ^cB^	
			(−14.87%)		(−9.11%)			(−33.87%)	
	30		30.28 ± 0.46 ^dB^		235.75 ± 6.22 ^dC^			184.78 ± 0.26 ^dB^	
			(−27.10%)		(−28.20%)			(−47.64%)	
	360		30.72 ± 0.76 ^dA^		266.14 ± 1.55 ^cA^			264.50 ± 15.75 ^bA^	
			(−26.05%)		(−18.95%)			(−25.04%)	
					
−20	0		44.91 ± 2.89 ^aAB^		328.85 ± 2.90 ^aB^			419.82 ± 2.89 ^aB^	
				
	7		31.95 ± 0.98 ^cC^		340.60 ± 31.77 ^aA^			294.98 ± 8.426 ^bB^	
			(−28.85%)		(+3.57%)			(−29.74%)	
	14		33.50 ± 0.81 ^cB^		227.64 ± 6.74 ^bB^			219.48 ± 1.75 ^dB^	
			(−25.41%)		(−30.78%)			(−47.72%)	
	30		39.12 ± 2.14 ^bA^		352.24 ± 14.69 ^aA^			235.47 ± 3.65 ^cdA^	
			(−12.90%)		(+7.11%)			(−43.91%)	
	360		30.57 ± 0.92 ^cA^		258.56 ± 17.23 ^bA^			270.27 ± 35.57 ^bcA^	
			(−31.94%)		(−21.37%)			(−35.62%)	

TPC: Total Phenolic Content; FRAP: Ferric-Reducing Antioxidant Power; TEAC: Trolox Equivalent Antioxidant Capacity; n.d.: non detected. Lowercase letters indicate statistically significant differences between samples stored at equal temperature for different storage times; capital letters indicate statistically differences among samples stored at different temperatures at equal storage time (one-way ANOVA followed by Tukey’s post hoc test; *p* < 0.05). The values in parentheses represent the percentage (%) variations compared with t_0_; negative and positive values indicate losses and gains, respectively.

## Data Availability

The original contributions presented in this study are included in the article/Supplementary Material. Further inquiries can be directed to the corresponding author.

## Supplementary Materials

The following supporting information can be downloaded at: https://www.mdpi.com/article/10.3390/antiox15030310/s1, Table S1: Multifactorial ANOVA analysis of the individual and interactive effects of storage temperature (T) and storage time (ST) on bitter acids, prenylflavonoids, polyphenols, total phenolic content and antioxidant capacity. Figure S1: Bitter acid content in hop cone samples after 360 days of storage at the different investigated temperatures. Figure S2: Prenylflavonoid content in hop cone samples after 360 days of storage at the different investigated temperatures. Figure S3: Polyphenol content in hop cone samples after 360 days of storage at the different investigated temperatures. Figure S4: CLMS micrographs of external and internal hop leaves stored for 360 days at −40 °C. Figure S5: CLMS micrographs of external and internal hop leaves stored for 360 days at −30 °C. Figure S6: CLMS micrographs of external and internal hop leaves stored for 360 days at −20 °C.
