# Supplementary material for: Unraveling the Effects of Freezing and Frozen Storage Temperatures on Hop Secondary Metabolites and Antioxidants
Source: Antioxidants (Basel). 2026 Feb 28;15(3):310. doi: 10.3390/antiox15030310 (PMC13023950; doi:10.3390/antiox15030310)
Supplement: Supplementary file 1 [file antioxidants-15-00310-s001.zip › antioxidants-4139111-Supplementary Table S1.pdf]

Table S1. Multifactorial ANOVA analysis of the individual and interactive effects of storage temperature (T) and storage time (ST) on bitter acids, prenylflavonoids, polyphenols, total phenolic content and antioxidant capacity

		F		
	**compound**	**T**	**ST**	**T×ST**
Bitter acids	Cohumulone	12.040***	211.916***	14.419***
	N+adhumulone	15.163***	208.551***	20.173***
	total α-acids	10.829***	149.794***	15.725***
	Colupulone	8.930***	95.905***	10.698***
	N+Adlupulone	12.023***	96.548***	11.624***
	total β-acids	10.825***	89.357***	11.160***
Preylflavonoids	Xanthohumol	26.766***	116.615***	9.165***
	Isoxanthohumol	n.s.	522.017***	2.637*
	8-Prenylnaringenin	164.29***	2326.39***	46.02***
Flavan-3-ols	Catechin	77.33***	394.49***	29.35***
	Epicatechin	14.15***	1013.72***	17.43***
Benzoic acids and derivatives	Gallic acid	139.00***	2496.14***	47.15***
	Syringic acid	16.45***	2220.33***	20.14***
	Vanillic acid	22.25***	1134.34***	15.29***
Flavanone	Naringenin	3.43*	219.65***	17.92***
Cinnamic acid and derivatives	Caffeic acid	27.5***	708.8***	35.1***
	Chlorogenic acid	17.51***	133.97***	2.45*
TPC		8.93***	170.64***	14.68***
FRAP		n.s.	75.79***	19.02***
TEAC		12.10**	237.98***	14.25***

TPC: Total Phenolic Content; FRAP: Ferric Reducing Antioxidant Power; TEAC: Trolox Equivalent Antioxidant Capacity, n.s. not significant. Significance level *$p < 0.05$; **$p < 0.01$; ***$p < 0.001$. T, temperature (°C), ST, storage time
