# Supplementary material for: Unraveling the Effects of Freezing and Frozen Storage Temperatures on Hop Secondary Metabolites and Antioxidants
Source: Antioxidants (Basel). 2026 Feb 28;15(3):310. doi: 10.3390/antiox15030310 (PMC13023950; doi:10.3390/antiox15030310)
Supplement: Supplementary file 1 [file antioxidants-15-00310-s001.zip › Supplementary figures captions.pdf]

SUPPLEMENTARY MATERIALS CAPTIONS

Figure S4. CLMS micrographs external (A, B, C) and internal (D, E, F) hop leaves stored for 360 days at −40°C. A1-2 and D1-2 represent merge of the channels ’blue-green’ and ‘red-orange’; B1-2 and E1-2, ‘blue-green’; C1-2 and F1-2 ‘red-orange’.

Figure S5. CLMS micrographs external (A, B, C) and internal (D, E, F) hop leaves stored for 360 days at −30°C. A1-2 and D1-2 represent merge of the channels ’blue-green’ and ‘red-orange’; B1-2 and E1-2, ‘blue-green’; C1-2 and F1-2 ‘red-orange’.

Figure S6. CLMS micrographs external (A, B, C) and internal (D, E, F) hop leaves stored for 360 days at −20°C. A1-2 and D1-2 represent merge of the channels ’blue-green’ and ‘red-orange’; B1-2 and E1-2, ‘blue-green’; C1-2 and F1-2 ‘red-orange’.
