# Supplementary figures and images for: Unraveling the Effects of Freezing and Frozen Storage Temperatures on Hop Secondary Metabolites and Antioxidants
Source: Antioxidants (Basel). 2026 Feb 28;15(3):310. doi: 10.3390/antiox15030310 (PMC13023950; doi:10.3390/antiox15030310)

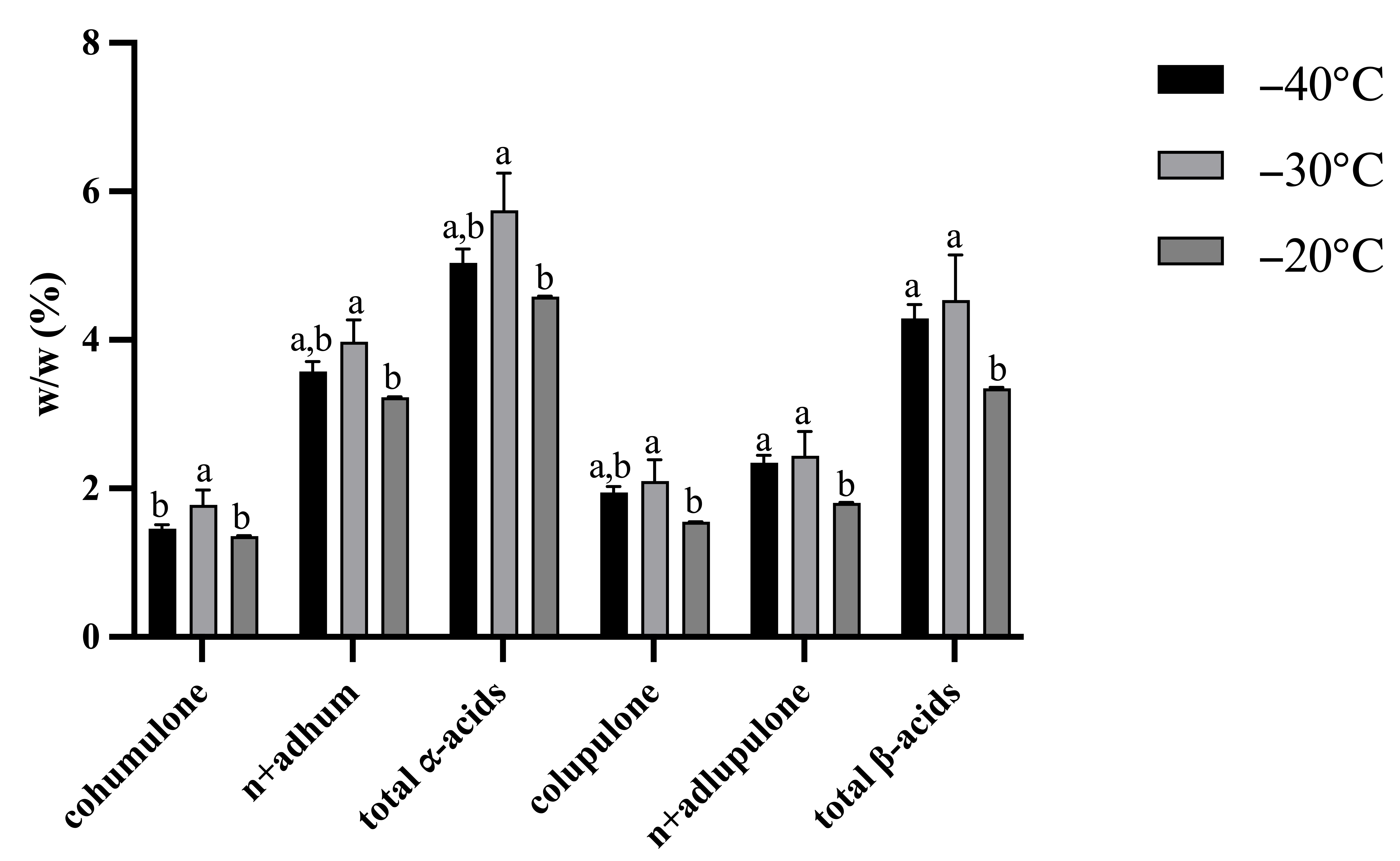

Supplement: Supplementary file 1 [file antioxidants-15-00310-s001.zip › Figure S1-bitter acids t360.png]

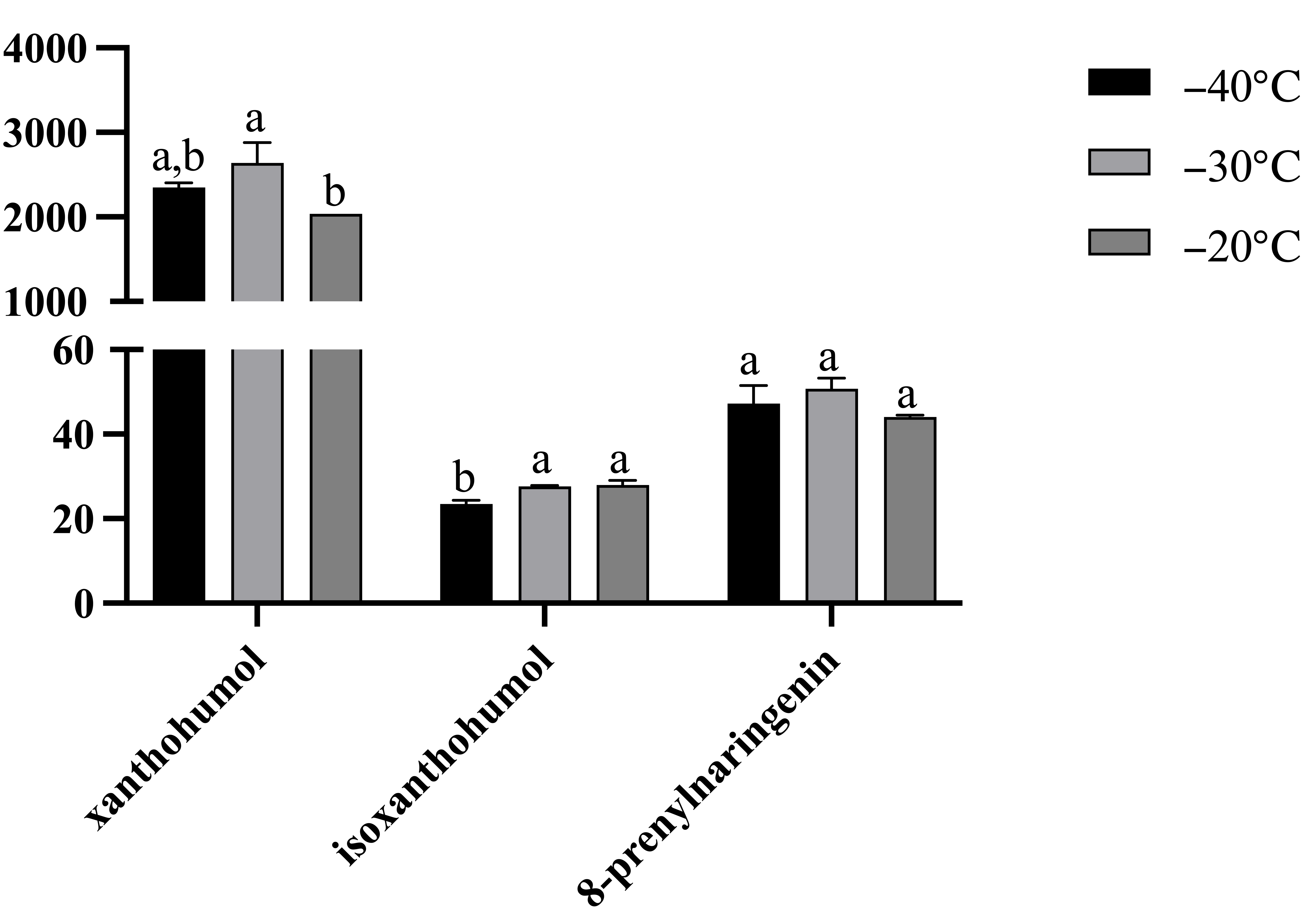

Supplement: Supplementary file 1 [file antioxidants-15-00310-s001.zip › Figure S2-prenylflavonoids-t360.png]

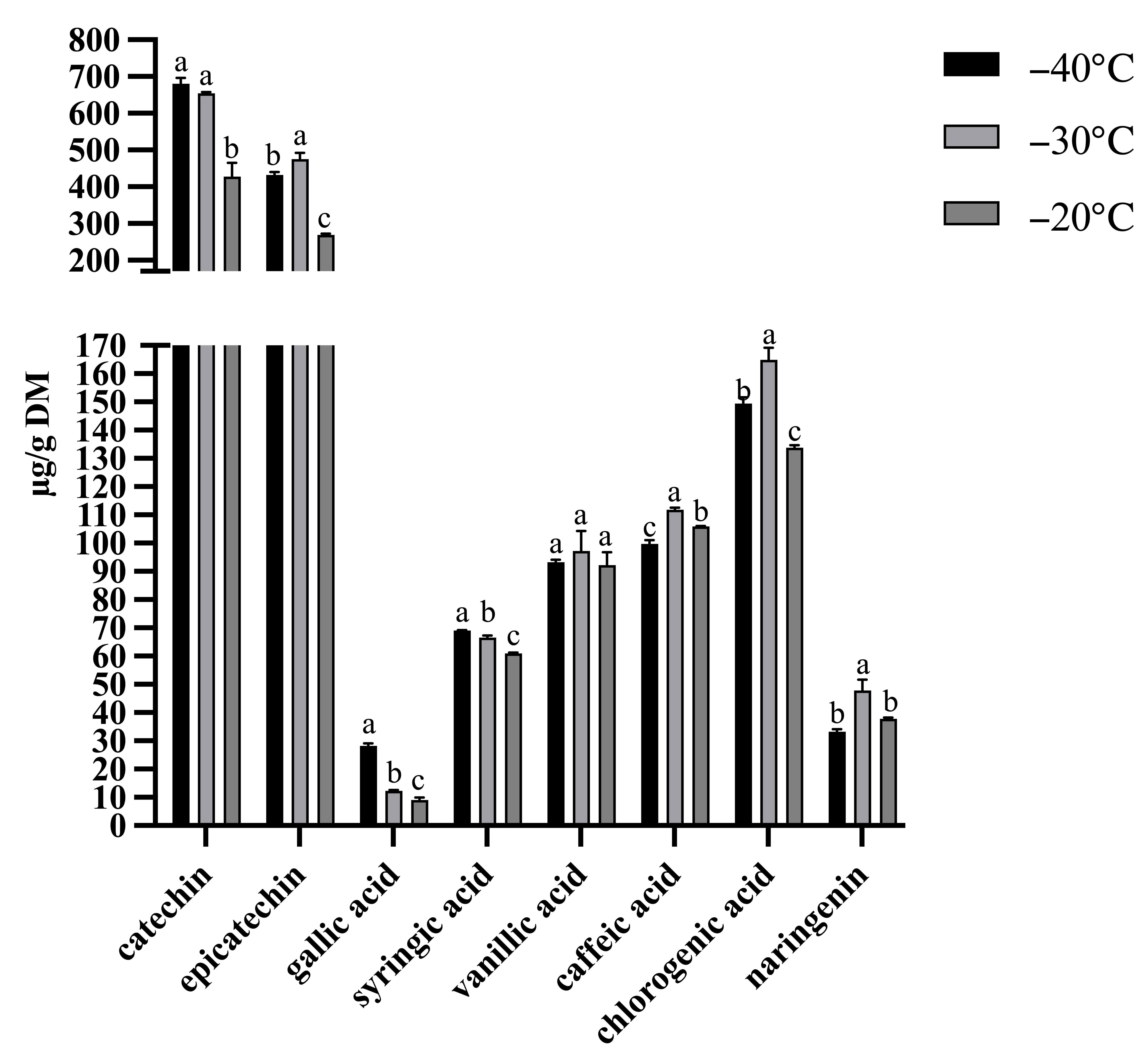

Supplement: Supplementary file 1 [file antioxidants-15-00310-s001.zip › Figure S3-polyphenols-t360.png]

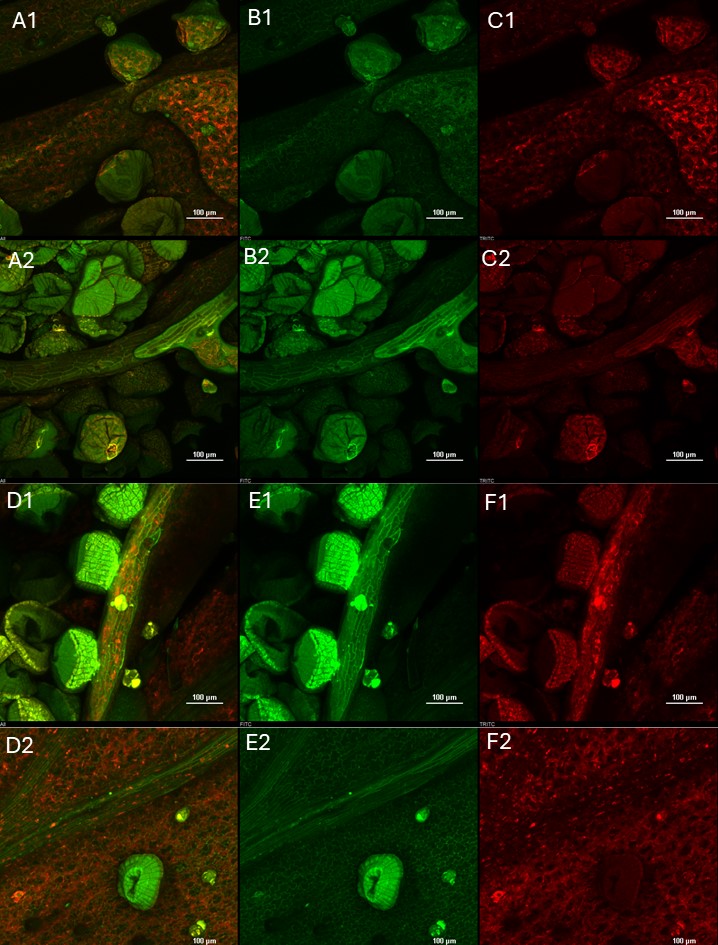

Supplement: Supplementary file 1 [file antioxidants-15-00310-s001.zip › Figure S4 CLSM.jpg]

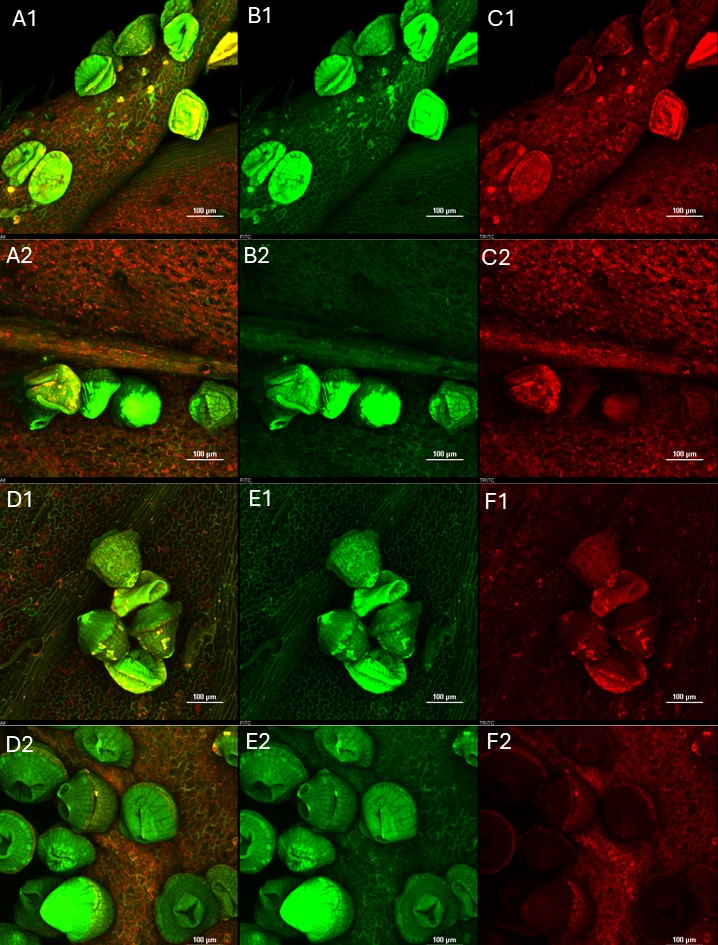

Supplement: Supplementary file 1 [file antioxidants-15-00310-s001.zip › Figure S5 CLSM.jpg]

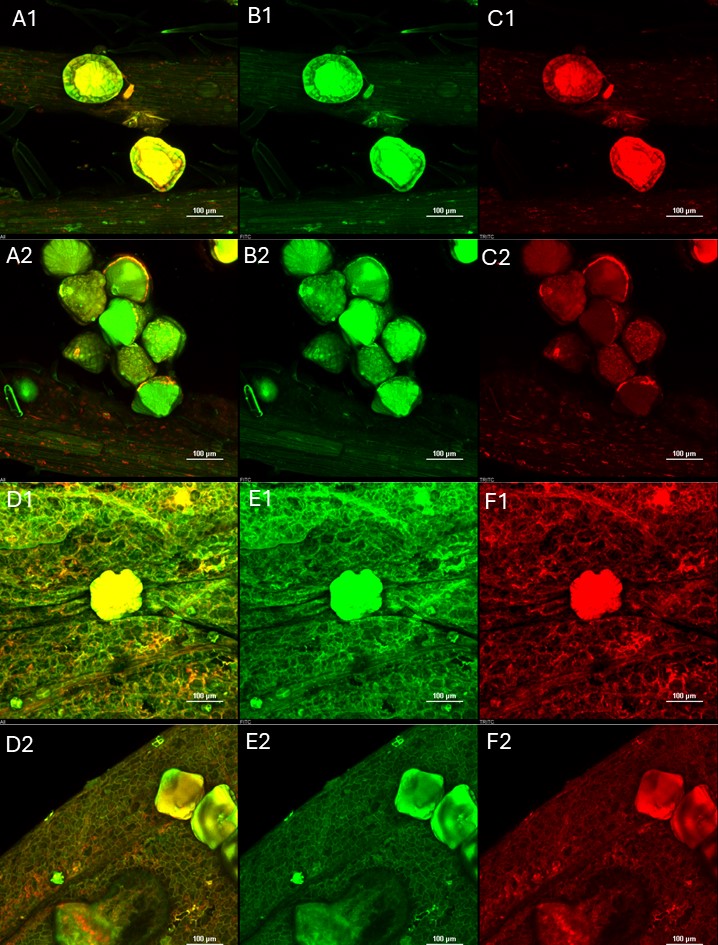

Supplement: Supplementary file 1 [file antioxidants-15-00310-s001.zip › Figure S6 CLSM.jpg]
